# What are the Metabolic Rates of Marine Mammals and What Factors Impact this Value: A review

**DOI:** 10.1093/conphys/coad077

**Published:** 2023-10-02

**Authors:** S R Noren, David A S Rosen

**Affiliations:** Institute of Marine Science, University of California Santa Cruz, Center for Ocean Health, 115 McAllister Way, Santa Cruz, CA 95060, USA; Marine Mammal Research Unit, Institute for the Oceans and Fisheries, University of British Columbia, 2202 Main Mall, Vancouver, BC, Canada V6T 1Z4

**Keywords:** Bioenergetics, field metabolic rate, basal metabolic rate, marine mammal, metabolism, modeling, mysticete, odontocete, pinniped, resting metabolic rate

## Abstract

Over the past several decades, scientists have constructed bioenergetic models for marine mammals to assess potential population-level consequences following exposure to a disturbance, stressor, or environmental change, such as under the Population Consequences of Disturbance (pCOD) framework. The animal's metabolic rate (rate of energy expenditure) is a cornerstone for these models, yet the cryptic lifestyles of marine mammals, particularly cetaceans, have limited our ability to quantify basal (BMR) and field (FMR) metabolic rates using accepted ‘gold standard’ approaches (indirect calorimeter via oxygen consumption and doubly labeled water, respectively). Thus, alternate methods have been used to quantify marine mammal metabolic rates, such as extrapolating from known allometric relationships (e.g. Kleiber's mouse to elephant curve) and developing predictive relationships between energy expenditure and physiological or behavioral variables. To understand our current knowledge of marine mammal metabolic rates, we conducted a literature review (1900–2023) to quantify the magnitude and variation of metabolic rates across marine mammal groups. A compilation of data from studies using ‘gold standard’ methods revealed that BMR and FMR of different marine mammal species ranges from 0.2 to 3.6 and 1.1 to 6.1 x Kleiber, respectively. Mean BMR and FMR varied across taxa; for both measures odontocete levels were intermediate to higher values for otariids and lower values of phocids. Moreover, multiple intrinsic (e.g. age, sex, reproduction, molt, individual) and extrinsic (e.g. food availability, water temperature, season) factors, as well as individual behaviors (e.g. animal at water’s surface or submerged, activity level, dive effort and at-sea behaviors) impact the magnitude of these rates. This review provides scientists and managers with a range of reliable metabolic rates for several marine mammal groups as well as an understanding of the factors that influence metabolism to improve the discernment for inputs into future bioenergetic models.

## Teaser

Assessing population-level consequences for marine mammals exposed to disturbance requires knowledge of their metabolism. We provide an overview of the magnitude and variation in marine mammal metabolism, and the intrinsic and extrinsic factors and behaviors that influence metabolism to assist with choosing a reliable metabolic rate for future bioenergetic models.

## The Need for Reliable Metabolic Rate Data

Over the past several decades, scientists have constructed bioenergetic models for marine mammals to assess potential population-level consequences of environmental changes and other stressors (reviewed in Pirotta, 2022). The animal's metabolic rate (rate of energy expenditure) is a cornerstone for these models. Bioenergetic models range from simple equations representing average energy expenditure, to detailed energy budgets for each age-class, sex and season, to complex dynamic models that incorporate changes in environmental conditions (Booth *et al.,* 2023). The reliability of the predictions that bioenergetic models provide are strongly dependent on the accuracy of the input variables (Enders and Scruton, 2006), which can be based upon laboratory and field experiments. The foundation of these models is the cost of running the animal—the animal’s metabolic rate—which represents the total energy expenditure (TEE) of an animal under a given set of circumstances.

Basal metabolic rate (BMR) and its associated measure resting metabolic rate (RMR) represent the baseline energy expenditure of an animal that includes the basic physiological costs associated with survival such as maintaining blood flow, cellular respiration and thermoregulation. BMR must be measured on animals under strict behavioral and physiological criteria: resting (awake but quiescent), postabsorptive, mature (non-growing) and non-reproductive (non-pregnant, non-lactating) and within its thermoneutral zone (Kleiber, 1975). Criteria for RMR are identical to those for BMR except that RMR may include measures obtained on immature or even reproductive animals. In comparison, field metabolic rate (FMR) is a broader term encompassing measures of energy expenditure of free-living individuals under a varied but individually well-defined set of circumstances. FMR is often considered the more ecologically relevant measure of energy expenditure because it includes the baseline energy consumption of the animal plus the additional energy required as the animal moves and survives in its habitat (i.e. energetic costs associated with feeding, locomoting, etc.; Nagy, 1994).

Marine mammals, depending on the group, live most if not all of their lives at sea, and the offshore lifestyles and prolonged submergence periods of some species (e.g. many species of beaked whales, sperm whales, *Physeter macrocephalus*) make it exceptionally difficult to measure their metabolism. For example, of the nearly 90 cetacean species, both odontocetes (toothed whales, dolphins and porpoises) and mysticetes (baleen whales), direct empirical measures of BMR have only been quantified in five odontocete species (Table 1) and direct measures of active metabolism have only been undertaken in three odontocete species (Table 2). Moreover, these studies are mostly restricted to smaller species that are readily maintained in aquaria. While there are more data from other groups of marine mammals (pinnipeds, mustelids, sirenians), measurements of FMR from wild individuals are often limited to times of the year when they are more readily accessible and hence may not be reflective of other seasons (reviewed by Davis, 2019). Yet ongoing human perturbations (e.g. climate change, disturbance from sound) are impacting food availability and disrupting foraging behaviors across seasons, making it increasingly important to have foundational knowledge about marine mammal metabolic rates in order to construct bioenergetic models that can make accurate assessments of population impacts (e.g. National Research Council, 2005; Costa, 2012).

**Table 1:** Mean basal metabolic rate (BMR) for each species expressed as a multiple of Kleiber's (1975) prediction for terrestrial mammals.

**Common Name**	**Species**	**Mean BMR as Multiple of Kleiber ± SD**	**References**
Beluga whale	*Delphinapterus leucas*	1.41 ± 0.39 (1.1–2.2)	Rosen and Trites (2013), John (2020)
Bottlenose dolphin	*Tursiops truncatus*	1.96 ± 0.45 (1.5–2.5)	Karandeeva *et al.* (1973), Feldkamp *et al.* (1987), Williams *et al.* (1993)Williams *et al.* (2001)van der Hoop *et al.* (2014), Williams *et al.* (2017), John (2020)
Killer whale	*Orcinus orca*	1.69 ± 0.78 (1.1–2.8)	Dunkin-McClenahan, unpub. Data; Kriete (1995)Worthy *et al.* (2014), Williams *et al.* (2017)
Pacific white-sided dolphin	*Lagenorhynchus obliquidens*	3.39 (n/a) (3.2–3.5)	Rechsteiner *et al.* (2013)
Harbor porpoise	*Phocoena phocoena*	2.45 (n/a)	Karandeeva *et al.* (1973)
**ODONTOCETE GRAND MEAN**		**2.18 ± 0.78**	
			
Australian fur seal	*Arctocephalus pusillus doriferus*	2.78 (n/a) (1.9–3.6)	Ladds *et al.* (2017c)
Australian sea lion	*Neophoca cinerea*	2.96 (n/a) (2.0–3.6)	Ladds *et al.* (2017c)
California sea lion	*Zalophus californianus*	2.72 ± 0.28 (1.9–3.8)	Liao (1990), Hurley and Costa (2001)
New Zealand fur seal	*Arctocephalus pusillus forsteri*	2.93 (n/a) (2.0–3.5)	Ladds *et al.* (2017c)
Steller sea lion	*Eumetopias jubatus*	3.64 ± 0.67 (3.0–5.7)	Fahlman *et al.* (2008), Goundie (2015), Fahlman *et al.* (2016a)
**OTARIID GRAND MEAN**		**3.01 ± 0.37**	
			
Bearded seal	*Erignathus barbatus*	1.03 (n/a)	Thometz *et al.* (2023)
Grey seal	*Halichoerus grypus*	1.26 ± 0.47 (0.7–2.3)	Fedak and Anderson (1982), Innes (1984), Lavigne *et al.* (1986), Boily and Lavigne (1995), Boily (1996), Boily and Lavigne (1997), Sparling *et al.* (2006)
Harbor seal	*Phoca vitulina*	1.37 ± 0.17 (01.1–1.6)	Matsuura and Whittow (1973), Innes (1984), Davis *et al.* (1985), Rosen and Renouf (1998)
Harp seal	*Pagophilus groenlandicus*	1.01 ± 0.22 (0.6–1.6)	Øritsland and Ronald (1975), Gallivan and Ronald (1979), Gallivan (1981), Innes (1984), Hedd *et al.* (1997)
Hawaiian monk seal	*Neomonachus schauinslandi*	1.17 (n/a)	John *et al.* (2021)
Ringed seal	*Pusa hispida*	1.13 ± 0.54 (0.6–2.0)	Parsons (1977), Innes (1984), Thometz *et al.* (2021)
Spotted seal	*Phoca largha*	1.69 (n/a) (1.5–1.9)	Thometz *et al.* (2021)
Weddell seal	*Leptonychotes weddellii*	1.87 ± 0.35 (1.6–2.3)	Kooyman *et al.* (1973), Castellini *et al.* (1992), Williams *et al.* (2001)
**PHOCID GRAND MEAN**		**1.32 ± 0.31**	
			
Walrus	*Odobenus rosmarus*	2.93 (n/a) (2.6–3.1)	Borque-Espinosa *et al.* (2021)
**ODOBENID GRAND MEAN**		**2.93 (N/A)**	
			
Sea otter	*Enhydra lutris*	2.54 ± 0.30 (2.2–3.0)	Morrison *et al.* (1974), Costa and Kooyman (1982), Costa and Kooyman (1984), Williams (1989), Yeates *et al.* (2007)
**MUSTELID GRAND MEAN**		**2.54 (n/a)**	
			
W. Indian manatee	*Trichechus manatus*	0.35 ± 0.19 (0.2–0.7)	Gallivan and Best (1980), Irvine (1983), John *et al.* (2021)
**SIRENIAN GRAND MEAN**		**0.35 (n/a)**	

Data are derived from studies of marine mammals that used indirect calorimetry (open-flow or pneumotachometer) to measure energy expenditure under conditions that satisfied Kleiber's original criteria. To calculate species means, a single average value from each source study was calculated, and these contributed equally to a species mean and standard deviation. The range (in parenthesis) was based on individual animal reported values. The grand mean for each taxonomic group of marine mammals is based on a single value for each included species. Individual data values are available in Appendix 1.

**Table 2:** Mean field metabolic rate (FMR) for each species expressed as a multiple of Kleiber's (1975) prediction for terrestrial mammals. Energy expenditure data are derived from studies of marine mammals that used indirect calorimetry after swimming on captive animals and wild Weddell seals (open-flow or pneumotachometer), or over a range of activities measured body mass changes of fasted wild animals or used doubly labeled water in wild animals and captive dolphins and porpoise.

**Common Name**	**Species**	**Mean FMR as Multiple of Kleiber ± SD**	**References**
Beluga whale	*Delphinapterus leucas*	3.01 (n/a) (2.4–4.1)	John (2020)
Bottlenose dolphin	*Tursiops truncatus*	4.18 ± 1.68 (2.1–7.2)	van der Hoop *et al.* (2014), Fahlman *et al.* (2016b), Bejarano *et al.* (2017), Williams *et al.* (2017), John (2020), Rimbach *et al.* (2021)
Harbor porpoise	*Phocoena phocoena*	3.36 (n/a/) 2.9–3.7	Rojano-Doñate *et al.* (2018)
**ODONTOCETE GRAND MEAN**		**3.52 ± 0.62**	
			
Antarctic fur seal	*Arctocephalus gazella*	4.37 ± 0.89 (3.3–5.5)	Costa and Trillmich (1988), Costa *et al.* (1989), Boyd and Duck (1991), Arnould *et al.* (1996), Jeanniard-du-Dot *et al.* (2016)
Australian fur seal	*Arctocephalus pusillus*	5.72 ± 0.79 (4.8–6.4)	Ladds *et al.* (2017a), Ladds *et al.* (2017c)
Australian sea lion	*Neophoca cinerea*	4.85 ± 0.98 (2.3–7.6)	Costa (1991), Costa and Gales (2003), Ladds *et al.* (2017a), Ladds *et al.* (2017c)
California sea lion	*Zalophus californianus*	5.52 ± 0.90 (2.9–6.9)	Costa (1986), Costa *et al.* (1991), McHuron *et al.* (2018)
Galapagos fur seal	*Arctocephalus galapagoensis*	1.07 (n/a) (0.7–1.8)	Costa and Trillmich (1988)
Galapagos sea lion	*Zalophus wollebaeki*	5.06 (n/a) (4.5–5.9)	Villegas-Amtmann *et al.* (2017)
New Zealand fur seal	*Arctocephalus pusillus forsteri*	6.06 ± 0.60 (2.2–9.3)	Ladds *et al.* (2017a), Ladds *et al.* (2017c)
Northern fur seal	*Callorhinus ursinus*	5.29 ± 0.81 (3.5–7.3)	Costa *et al.* (1985), Costa and Gentry (1986), Jeanniard-du-Dot *et al.* (2016), McHuron *et al.* (2019)
Steller sea lion	*Eumetopias jubatus*	4.97 ± 1.50 (3.7–6.6)	Goundie *et al.* (2015), Ladds *et al.* (2017a)
**OTARIID GRAND MEAN**		**4.73 ± 1.57**	
			
Grey seal	*Halichoerus grypus*	3.24 ± 1.99 (1.3–4.6)	Anderson and Fedak (1985), Sparling *et al.* (2008)
Harbor seal	*Phoca vitulina*	3.76 ± 1.65 (2.3–6.0)	Davis *et al.* (1985), Reilly and Fedak (1991), Bowen *et al.* (1992), Coltman *et al.* (1998)
Hawaiian monk seal	*Neomonachus schauinslandi*	3.17 (n/a) (2.8–3.5)	John (2020)
Hooded seal	*Cystophora cristata*	4.22 (n/a)	Kovacs *et al.* (1996)
Northern elephant seal	*Mirounga angustirostris*	1.19 ± 0.11 (1.0–1.5)	Maresh (2014), Maresh *et al.* (2014)
Weddell seal	*Leptonychotes weddellii*	2.17 ± 1.18 (1.3–4.5)	Kooyman *et al.* (1973), Kooyman *et al.* (1980), Kooyman *et al.* (1983), Bartsh *et al.* (1992), Castellini *et al.* (1992), Ponganis *et al.* (1993)
**PHOCID GRAND MEAN**		**2.96 ± 1.11**	
			
Walrus	*Odobenus rosmarus*	5.28 ± 1.91 (2.8–7.2)	Acquarone *et al.* (2006), Rosen (2020), Borque-Espinosa *et al.* (2021)
**ODOBENID GRAND MEAN**		**5.28 (n/a)**	
			
Sea otter	*Enhydra lutris*	4.49 (n/a)	Yeates *et al.* (2007)
**MUSTELID GRAND MEAN**		**4.49 (n/a)**	

To calculate species means, a single average value from each source study was calculated, and these contributed equally to a species mean and standard deviation. The range (in parenthesis) was based on individual animal reported values. The grand mean for each taxonomic group of marine mammals is based on a single value for each included species. Individual data values are available in Appendix 2.

## Methods for Estimating Metabolic Rate

In theory, metabolism is most directly measured as heat production (direct calorimetry), an essentially impossible task to perform with marine mammals. Hence, there are several accepted alternate standards for measuring metabolism in marine mammals that are considered ‘gold standards’. BMR (and RMR) is usually measured via respirometry (a form of indirect calorimetry), using an apparatus that quantifies the rate of oxygen consumption and carbon dioxide (CO_2_) production that is then converted to rates of energy consumption. Meanwhile, FMR is usually measured via the turnover of water containing two different isotopes of hydrogen and oxygen injected into the animal, known as the doubly labelled water (DLW) method; this yields an estimate of CO_2_ production that is converted into rates of energy consumption (reviewed in Iverson *et al.,* 2010). Older studies used changes in body mass to estimate FMR, but this technique can only be applied to fasting animals where all energy requirements are met through tissue catabolism. Another group of studies have measured the cost of subsurface swimming, usually using respirometry on animals in aquaria. For the purposes of our review (detailed below), we have included all of these measures as estimates of FMR.

Due to the logistical difficulties of measuring energy expenditure in marine mammals through the aforementioned methods, researchers have attempted to quantify metabolic rates using various different approaches. Alternate methods have included the development of predictive relationships between metabolic rate and physiological (e.g. heart rate, respiration rate, caloric intake) or behavioral (e.g. swim speed, measures of body movement or acceleration) characteristics (Williams *et al.,* 1993; Boyd *et al.,* 1999; Butler *et al.,* 2004; Fahlman *et al.,* 2008; Young *et al.,* 2011; Dalton *et al.,* 2014; Noren *et al.,* 2014; Kastelein *et al.,* 2018; McHuron *et al.,* 2022). Unfortunately, there are inherent inaccuracies when using such proxies due to unvalidated or weak relationships and substantial variation between individuals (McPhee *et al.* 2003). For example, the accuracy of using respiratory rate as a proxy for metabolic demand, such as when applied to captive beluga whales (*Delphinapterus leucas*; George and Noonan, 2014), is unknown when variation in breathing volume and duration is not taken into account (Roos *et al.,* 2016; Fahlman *et al.,* 2016b). Although heart rate was correlated with energy expenditure in some otariids (Butler *et al.,* 1992; Boyd *et al.,* 1995), it was not accurate for quantifying energetics of individual dives (Young *et al.,* 2011). Measures of animal movement through their environment, such as overall dynamic body acceleration (ODBA), are theorized to be related to energy expenditure (Halsey *et al.,* 2009). However, ODBA has been shown to poorly predict measured energy expenditure in a number of marine mammal species (Volpov *et al.,* 2016; Ladds *et al.,* 2017a; Pagano and Williams, 2019) due to the effects of post-exercise recovery, stress, thermoregulation, specific dynamic action, reproduction and growth (Wilson *et al.,* 2020). Overall, each of these proxy methods comes with its own list of assumptions and inherent errors, and therefore we do not include them in our review as we attempt to answer the question, ‘What are the energy requirements of marine mammals?’

**Figure 1:**
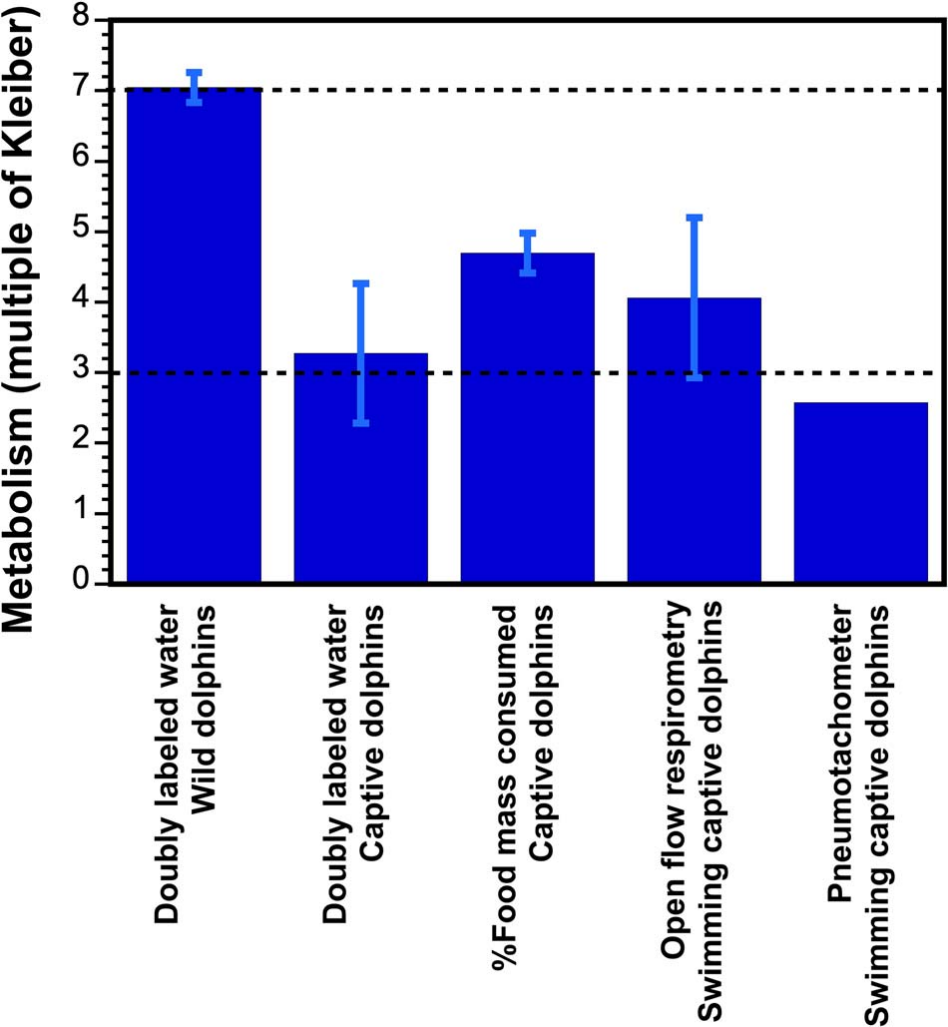
Empirical measurements of field metabolic rate (FMR) in the bottlenose dolphin (*Tursiops truncatus*) using different methodologies (bars). Data are presented for studies that used doubly labeled water to measure total energy expenditure (TEE) of wild dolphins (Bejarano *et al.,* 2017) and captive dolphins (Rimbach *et al.,* 2021), estimates based on food intake as a proportion of body mass of captive dolphins (Bejarano *et al.,* 2017) and studies on captive dolphins where metabolic rate was measured after subsurface swimming using either open flow respirometry (van der Hoop *et al.,* 2014; Williams *et al.,* 2017; John, 2020) or a pneumotachometer (Fahlman *et al.,* 2016b). Bars represent mean FMR ± standard deviation across reported FMR values and are expressed as a multiple of Kleiber (data in Appendix 2). Horizontal dashed lines illustrate the general range of estimates for FMR (between 3-7x Kleiber) that have been traditionally reported in the literature for various marine mammals, as illustrated in Costa and Maresh (2018).

Even when using accepted ‘gold standard’ approaches to measure metabolism there can be variations in the value reported due to differences in methodology, as well as testing regime. This is evident when comparing estimates of FMR for bottlenose dolphins (*Tursiops truncatus*) that have used a variety of methods (Figure 1). Even when using a single technique, such as respirometry to measure either BMR or swimming metabolism, it is important to regulate the animal's behaviors during and prior to data collection. This is because animal behavior (including psychological stress level) has a large influence on metabolism, and daily living activity can have residual effects on the metabolic measurement. Thus, it is often recommended that subjects must rest a minimum of 20 minutes prior to the start of the experiment, while moderate to vigorous activity must be controlled for hours before the test as it can have an even longer carryover effect (Compher *et al.*, 2006). Although arbitrarily extending the measurement period does not necessarily increase the accuracy of BMR measurements (Reeves *et al.,* 2004), it has been recommended that, to obtain accurate resting metabolic measurements, the test duration should be 10 minutes in duration with the first 5 minutes discarded, and the remaining 5 minutes averaged; and these 5 minutes are only averaged if there is a coefficient of variation < 10% (Compher *et al.*, 2006). While this approach is not possible with a one breath sample from a pneumotachometer, studies report that results obtained by this method are similar to those from traditional respirometry (Allen *et al.,* 2022). In addition to these methodological constraints, there are a number of technical and analytical procedures that must be carefully followed in order to obtain meaningful estimates of energy expenditure from respirometry (Lighton, 2008).

Although the use of DLW for estimating FMR is well established (Lifson and McClintock, 1966), the results are also impacted by subtle differences in methodology (Speakman and Krol, 2005). For example, the calculated metabolic rate reported will depend on which specific mathematical equation was used to convert chemical turnover to energy expenditure (e.g. compare Nagy, 1980, Speakman *et al.,* 1993). Moreover, despite the ability of DLW to provide estimates of energy expenditure in free-ranging animals, it is difficult to plug these values into a bioenergetic model because they represent mean energy expenditure over a period that includes numerous types of behaviors (Skinner *et al.,* 2014), and these behaviors will vary temporarily and spatially within and across individuals and species. For example, measurements of FMR in lactating female pinnipeds will vary significantly depending if the time the animal spent on land was included or if only the time spent at sea is considered (Costa *et al.,* 1991).

Given the difficulties of measuring marine mammal metabolism, it is tempting to extract the required values from previously published allometric equations. For example, one might use Kleiber’s (1975) equation predicting BMR of terrestrial mammals from body mass to estimate the energy expenditure of marine mammals, since it has been long-established that body size accounts for the majority of the variation in metabolic demand in mammals (Benedict, 1938; Kleiber, 1975; Nagy, 2005). However, there are considerable lifestyle, ecological and taxonomic factors that significantly alter the exact relationship between body mass and measures of energy expenditure (Glazier, 2005). Thus, using a curve based on terrestrial mammals could result in erroneous conclusions when marine mammals have numerous adaptations to a life in water that would complicate such extrapolations. Indeed, as discussed further below, some studies suggest that marine mammals have elevated BMR compared with terrestrial mammals of similar size. Hypothesized reasons for an elevated BMR include the increased cost of staying warm in water, a medium with much higher thermal capacity and conductivity than air (South *et al.,* 1976, Costa and Kooyman, 1984, Costa and Williams, 1999, Williams, 1999), and the consequences of a carnivorous diet (Williams *et al.,* 2001). Conversely, the amount of blubber stored by marine mammals to stay warm could complicate the scaling of metabolism with body size since fat is largely metabolically inactive (Sparti *et al.,* 1997). For example, lean mass was found to be a better predictor of BMR than total body mass in adult harp seals (*Pagophilus groenlandicus*; Aarseth *et al.,* 1999) and RMR in northern elephant seal pups (*Mirounga angustirostris*; Rea and Costa, 1992). In addition, the body mass of larger-bodied cetaceans is well beyond the predictive ‘mouse to elephant curve’ (Kleiber, 1975); it would be statistically erroneous to extrapolate the curve beyond the range of body mass data presented.

## Are Metabolic Rates of Marine Mammal Elevated Compared to Equally Sized Terrestrial Mammals?

The question of whether marine mammals have elevated metabolic rates compared to their counterparts has been debated for decades. Its importance is heightened by the potential impact of marine mammal predation on fisheries resources (Matthiopoulos *et al.,* 2008). Past reviews of the literature do not provide a consensus about the magnitude of marine mammal metabolic rates. Some reviews conclude that BMR of marine mammals are not significantly different from terrestrial counterparts (Lavigne *et al.,* 1986; Boyd, 2002; Hunter, 2005), while others conclude the opposite (Williams *et al.,* 2001; Davis, 2019). Several authors similarly suggest that most marine mammal groups have elevated FMRs. A review by Williams *et al.* (2020) indicated that the FMRs of most marine mammal groups are about 1.3 times higher than similarly sized terrestrial carnivores, a finding similarly reported by Rimbach *et al.* (2021) who also noted that there was substantial overlap between the two groups. Meanwhile, others have suggested that the scaling exponent for the relationship between body mass and FMR in most marine mammal groups is lower than that for terrestrial mammals (Boyd, 2002; Maresh, 2014; Costa and Maresh, 2018). This would mean that the field energy expenses of the smallest marine mammals are higher than predicted compared to equivalent terrestrial mammals, while the energy expenditures of larger marine mammals (>250 kg) are lower than expected (Boyd, 2002; Maresh, 2014). This relationship may exist because once a marine mammal is large enough their low surface-area to volume ratios minimize heat loss to the environment and their overall maintenance costs are lower than that of terrestrial mammals given that they do not use energy to support their body weight due to the buoyant force of water (Innes, 1986; Boyd, 2002).

Given the diverse views on whether marine mammals have elevated metabolic rates compared to equally sized terrestrial mammals, we should revisit the difficulties surrounding measuring BMR of marine mammals. In their review, Lavigne *et al.* (1986) noted that some studies of marine mammal metabolism did not conform to the standardized measurement criteria as defined by Kleiber (1975), and when such ‘Kleiber conditions’ are not met the measured metabolic rate (almost by definition) can be higher. In addition to inappropriate measures on immature and reproductive individuals, one common issue is that food rewards may be used as an incentive for obtaining metabolic measures from trained animals, which will have a significant effect on metabolism ([Bibr ref43][Bibr ref43], Barbour, 1993, Rosen and Trites, 1997). For example, the metabolism of juvenile South American fur seals (*Arctocephalus australis*) increased by 63% from the post absorptive to postprandial condition (Dassis *et al.,* 2014) and harbor porpoises (*Phocoena phocoena*) showed a 1.3 increase in metabolism after food consumption (Reed *et al.,* 2000).

## What Are the Metabolic Rates of Marine Mammals if We Only Consider ‘Gold Standard’ Measurements

Given the long-standing debate over whether marine mammal metabolism is higher compared to terrestrial mammals, and with the knowledge that some methods to quantify metabolism are not as reliable as others, we compiled the metabolic data published for marine mammals from 1900 to 2023 that used the ‘gold standard’ methods. For BMR, we only considered measures obtained via indirect calorimetry; either open-flow respirometry via a dome or pneumotachometer, plus a few early studies using closed breathing or mask systems. While the usual standard for FMR are measures obtained via DLW, due to limited number of such studies from cetaceans we also included empirical studies that provided FMR based on swimming metabolic rate (obtained via respirometry), as well as FMR based on mass loss in fasting animals (predominantly pinnipeds).

Several previous studies have similarly compiled available data (Lavigne *et al.,* 1986; Boyd, 2002; Hunter, 2005; Maresh, 2014; Davis, 2019), and our review included careful re-examination of whether the BMR values met required, standard conditions (i.e. fasted, thermal neutral zone, resting but not sleeping, mature but non-reproductive at the time of the study). We also endeavored to record data at the level of the individual animal rather than just reporting the mean from each study or an overall species mean, although this information was not always provided in the original papers. In addition, we tried to ensure that identical data points that have been reported across multiple publications were not duplicated in our database. In a few cases, there were data from multiple studies that used the same study animals; these were included in our database. We also noted the testing medium (water or air) and the methodology used (e.g. open-flow respirometry vs. pneumotach). For FMR measures, we noted the methodology (e.g. DWL, respirometry, body mass changes) and life history/behavioral stage represented during the study (e.g. lactating female vs at-sea portion only vs fasting breeding male, swimming, etc.). Only FMR data from adult animals were used, to make them partially comparable to collated BMR values.

To compare across species, we standardized the reported rates of energy expenditure into kcal per day (kcal d^−1^) from various reported units of energy expenditure (e.g. kJ∙d^−1^, Watts per hour, mL O_2_ per min, etc.) using accepted conversions. Although we report values in kcal, this can easily be converted to MJ with the conversion of 1 MJ equivalent to 238.85 kcal. These rates of absolute energy expenditure were then converted to mass-specific measures to allow us to standardize across marine mammal species that represent a large range in body mass. Although there is no consensus on how to account for inter- and intra-specific differences in body mass (Hochachka *et al.,* 2003), we chose to express metabolic rates on a mass-specific basis (kcal d^−1^ per kg). This process was necessary, but unfortunately, it reduced the number of studies that could be included in our analyses since several studies did not provide the mass of their subjects. We then converted these absolute values to multiples of that predicted from Kleiber’s (1975) ‘mouse-to-elephant’ equation for RMR of terrestrial mammals (70 * Mass^0.75^ kcal d^−1^) for those that wish to revisit the long-standing debate about whether marine mammals have elevated metabolic rates compared to their terrestrial counterparts.

**Figure 2:**
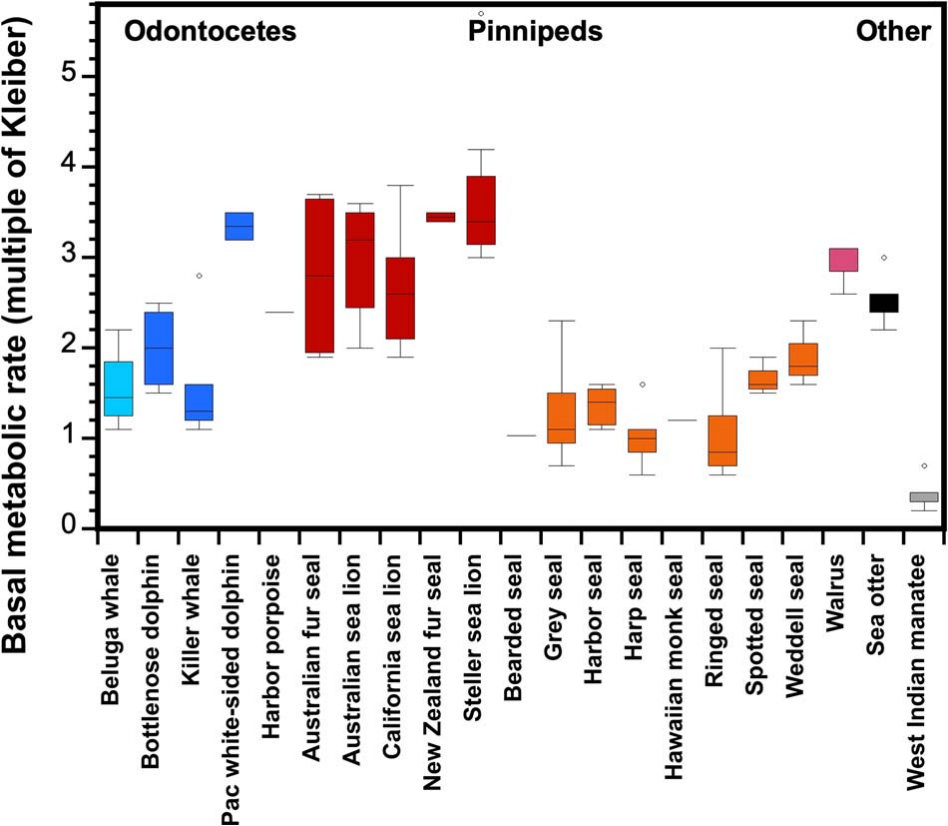
Basal metabolic rate (BMR) represented as a multiple of Kleiber’s (1975) equation for various species of marine mammals. Box plots (including statistical outliers represented as circles) are based upon individual data provided in Appendix 1, which contains details on the methods used and the original references. This graph only includes data that satisfied all of the physiological conditions for “Kleiber condition” BMR and used one of the “gold standard” approaches for measuring BMR (respirometry using a dome, mask, or pneumotachometer). Within the Odontocetes, different shades of blue differentiate the families Monodontidae (beluga whale; light blue), Delphinidae (bottlenose dolphin, killer whale, Pacific white-sided dolphin; medium blue) and Phocenidae (harbor porpoise; dark blue). Similarly, pinnipeds are grouped by family Otariidae (Australian fur seal, Australian sea lion, California sea lion, New Zealand fur seal, Steller sea lion; red), Phocidae (bearded seal, gray seal, harbor seal, harp seal, Hawaiian monk seal; orange), or Odobenidae (walrus; pink). Data for the sea otter (black) and West Indian manatee (grey) are also provided.

**Figure 3:**
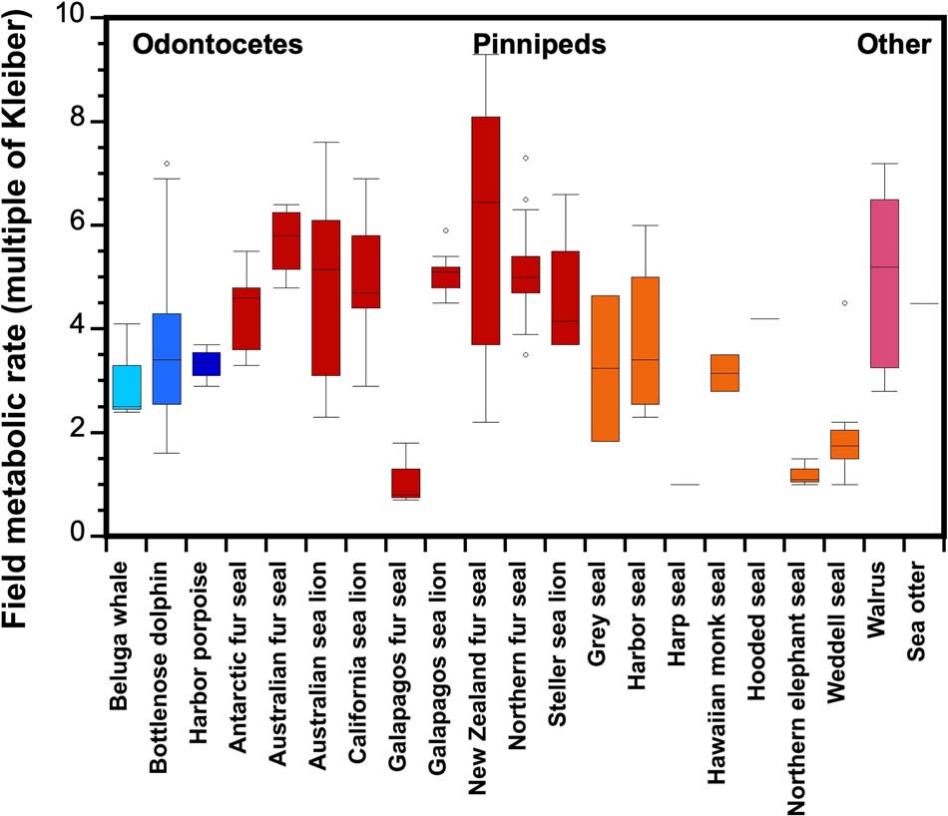
Field metabolic rate represented as a multiple of Kleiber's (1975) equation for various species of marine mammals. Box plots (including statistical outliers represented as circles) are based upon individual data provided in Appendix 2, which contains details on the methods used and the original references. Within the Odontocetes, different shades of blue differentiate the families Monodontidae (beluga whale; light blue), Delphinidae (bottlenose dolphin; medium blue) and Phocenidae (harbor porpoise; dark blue). Similarly, pinnipeds are grouped by family Otariidae (Antarctic fur seal, Australian fur seal, Australian sea lion, California sea lion, Galapagos fur seal, Galapagos sea lion, New Zealand fur seal, Northern fur seal, Steller sea lion; red), Phocidae (gray seal, harbor seal, harp seal, Hawaiian monk seal, hooded seal, Northern elephant seal, Weddell seal; orange), or Odobenidae (walrus; pink). Data for the sea otter (black) are also provided.

These individual converted data appear in Appendix 1 (BMR) and Appendix 2 (FMR). From these data, we calculated the mean for each species within each study. From those means we calculated an overall mean for the species, which are provided in Table 1 (BMR) and Table 2 (FMR); in this way, each study (regardless of number of measures) contributed equally to the species mean. Finally, from the appropriate species means, we calculated grand taxa means for odontocetes, sirenians, phocids, odobenids and mustelids. The results confirm that marine mammals are not a homogenous group. The range of BMRs (Appendix 1; Table 1; Figure 2) and FMRs (Appendix 2; Table 2; Figure 3) varied across individuals, species and marine mammal taxa.

Our analysis revealed that there are differences in the baseline energy needs across the three families of pinnipeds: Phocidae (‘true seals’), Otariidae (sea lions and fur seals) and Odobenidae (walruses). Within this taxa, phocids had the lowest grand mean BMR (1.32 ± 0.31 x Kleiber), with species means ranging from 1.0 for the harp seal (*Pagophilus groenlandicus*) to 1.9 for the Weddell seal (*Leptonychotes weddellii*). The latter may be an over-estimate, as the measures were done at naturalistic breathing holes and it was unclear in many of the studies whether foraging had occurred. It should also be noted that across marine mammals, the phocids had the largest dataset with data for 7 of 19 extant species. Interestingly, the closely related otariids had the highest grand mean BMR (3.01 ± 0.37) among all marine mammal taxa. There was a high level of consistency among four of the five otariid species that have been studied, where RMR ranged from 2.7 to 3.0 x Kleiber. The BMR (3.6 x Kleiber) of the fifth species, the Steller sea lion (*Eumetopias jubatus*), was notably higher than the others; this result may, in part, be due to high BMRs in a few of the studies (Appendix 1). Meanwhile, there is only a single published study for a mature odobenid, the walrus (*Odobenus rosmarus*; Borque-Espinosa *et al.,* 2021). They reported a relatively high BMR (2.9 x Kleiber) compared to the other marine mammals, and this value was curiously higher than the RMR (1.9 x Kleiber) reported for two juvenile walrus (Rosen, 2020). As noted by Borque-Espinosa *et al.*(2021), the elevated BMR in the adult walrus may have been related to the animal being somewhat active during testing.

As mentioned previously in the text, it has been extremely difficult to measure the BMR of cetaceans. As a result, only five species of odontocetes have been studied to date. These data represent species from three families, Monodontidae (one species), Delphinidae (three species) and Phocoenidae (one species). The grand mean BMR across these odontocetes was 2.18 ± 0.78 x Kleiber, which is intermediate to the grand means reported for the pinniped families. Closer inspection reveals that the Arctic dwelling monodontid (beluga whale) has the lowest BMR (1.41 ± 0.3 x Kleiber) amongst the odontocete species measured to date. Next are two of the three delphinids, killer whale (*Orcinus orca*) at 1.69 ± 0.45 x Kleiber and bottlenose dolphin at 1.96 ± 0.45 x Kleiber, followed by the harbor porpoise at 2.45 x Kleiber and the third delphinid (Pacific white-sided (PWS) dolphin; *Lagenorhynchus obliquidens*) at 3.39 x Kleiber. The harbor porpoise and PWS are comparatively small odontocetes that live in cold temperate waters; thus, an intrinsically high metabolism may be required for thermal neutrality.

Finally, of all the marine mammals, the lowest BMR (0.35 x Kleiber) is found in the sirenians (represented by the West Indian manatee, *Trichechus manatus*). This low BMR is likely related to their herbivorous and sedentary lifestyle in a warm-water environment. Surprisingly, the BMR (2.54 x Kleiber) for Mustelids represented by the sea otter (*Enhydra lutris*), is intermediate to that of odontocetes and otariids. This is an interesting finding since numerous studies have suggested that sea otters, as the smallest marine mammal and the only marine mammal without a blubber layer, must rely on intrinsically high BMR to maintain thermal neutrality (e.g. Yeates *et al.,* 2007). Alternatively, sea otters may rely on thermal substitution from digestion (heat increment of feeding) or activity to maintain thermoneutrality (Costa and Kooyman, 1984).

Unfortunately, it is extremely difficult to obtain direct measures of BMR from large cetaceans. Indeed, there are no respirometry estimates of BMR available for mysticetes, as their large size typically precludes even temporary holding. Wahrenbrock *et al.* (1974), however, used a combination of captured breath samples and respiration rates to measure the resting metabolism of two gray whale calves (*Eschrichtius robustus*), with estimates ranging from 0.9 to 2.4 x Kleiber. Estimating metabolic expenditure from respiration characteristics has been explored by several researchers, particularly for larger odontocetes (Roos *et al.,* 2016; Fahlman *et al.,* 2016b; Nazario *et al.,* 2022). Alternate approaches for estimating resting energy expenditure of odontocetes from physiological data have included pulmonary mechanics in pilot whales, *Globicephala scammoni* (6.4 x Kleiber; Olsen *et al.,* 1969) and heat exchange thermodynamics in Hawaiian spinner dolphin, *Stenella longirostris* (1.5 x Kleiber; Hampton and Whittow, 1976).

Although there are more estimates of FMR than BMR among marine mammals, there is less consistency in what these values represent. As discussed later, the magnitude of FMR measures is highly dependent upon the type of behavior captured. Still, there is value in comparing across broad taxonomic and behavioral categories. Like BMR, we find differences in FMR across the 3 families of pinnipeds (Table 2). Also like BMR, the lowest FMR across the pinnipeds was found in phocids, where the grand mean of six phocid species was 2.96 ± 1.11 x Kleiber, and ranged from 1.2 to 4.2 x Kleiber across the species. Meanwhile, the grand mean FMR of nine species of otariids was 4.73 ± 1.57 x Kleiber (range, 1.1–5.7 x Kleiber) and the mean FMR for odobenids (represented by walrus) was 5.28 ± 1.91 x Kleiber (range, 2.8–7.2 x Kleiber). Interestingly, the FMR for the sea otter (4.49 x Kleiber) is intermediate to the grand means provided for the three pinniped families, similar to the trend seen in BMR. However, it should be noted that this value was derived from a single study that combined captive measurements of behavior-specific energy expenditure and behavioral budgets of wild male otters and, therefore, does not represent a direct measure of FMR (Yeates *et al.,* 2007).

To date, the FMR of cetaceans has only been measured in three odontocete species. As with BMR, the grand mean FMR for odontocetes (3.52 ± 0.62 x Kleiber) is intermediate to the values reported for the pinniped families (Table 2). The FMR means across odontocete species ranged from 3.0 to 4.2 x Kleiber, with the Arctic dwelling beluga whale having the lowest FMR, followed by the harbor porpoise, and lastly, the bottlenose dolphin (Table 2). It is curious that the mean FMR for the bottlenose dolphin is higher than the mean FMR for the harbor porpoise, considering that the bottlenose dolphin mean BMR was lower than that of the porpoise. Looking at the FMR values from each study, we find that the range of reported FMR values was wider for the bottlenose dolphin (Table 2), which could be because different methodologies and types of measurements were used across the bottlenose dolphin studies (Appendix 2). While DLW estimates of total energy expenditure in captive dolphins were similar to the costs of short-term submerged swimming measured via respirometry in captive dolphins, TEE estimates using DLW from wild dolphins were approximately 2 times greater (Fig. 1). Given the lack of empirical FMR data for mysticetes, some researchers have tried to produce predictive equations derived from other cetaceans based solely upon body mass. However, as just discussed, treating all marine mammal taxa similarly will likely lead to erroneous estimates.

## What Is the Relationship between Different Measures of Metabolism in Marine Mammals?

**Figure 4:**
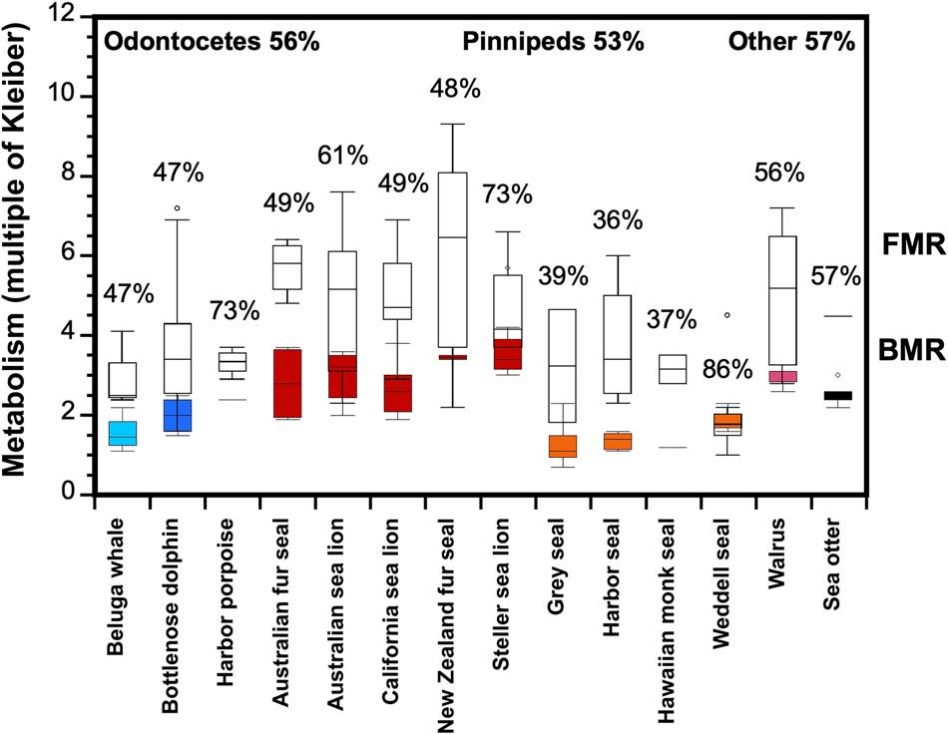
Basal metabolic rate (BMR) in comparison to field metabolic rate (FMR) for various species of marine mammals expressed as a multiple of Kleiber’s (1975) equation. The box plots representing BMR (colored box plots) and FMR (clear box plots) are based on individual study means for each species, as shown in Fig. 2 and Fig. 3, respectively. The calculations for BMR as a proportion of FMR are shown as percentages and are based on species means detailed in Table 1 and Table 2 (which includes references for the original data). The grand mean of BMR as a proportion of FMR (%) across all odontocetes species and across all pinniped species are shown in bolded font. The value for “Other” solely represents the sea otter.

One of the reasons measures of BMR (and RMR) are considered valuable for studying the energetics of animals is that they are often assumed to be related to daily energy expenditure (Carpenter *et al.,* 1995; Johnson *et al.,* 2001; Burton *et al.,* 2011). Therefore, we were curious to know what proportion of FMR was attributable to maintenance costs (BMR) for these animals. From our review of the literature, it was evident that BMR has been measured in many more marine mammal species than FMR (Appendix 1 and 2). However, if there is a reliable relationship between RMR and FMR, it may be possible to use some multiple of BMR to estimate FMR for species without empirical measures. Admittedly, the paucity of FMR studies limits this analysis (especially for cetaceans). We also acknowledge that is it not ideal to combine data across studies, where the animals varied in age, sex, reproductive status and satiation and exhibited different behaviors, and experienced different conditions like time of day, season, air and water temperature. Yet despite these limitations, the initial findings of this type of analysis are intriguing. Broadly, there was similarity across taxa, which was surprising since evolutionarily, cetaceans, pinnipeds and mustelids were not derived from a common terrestrial ancestor. Across three odontocete species, BMR represented 56% of FMR, which was similar in value to that found for the three pinniped families, phocid seals (50%; four species), otariids (56%; five species) and walrus (56%), as well as the mustelid the sea otter (57%; Figure 4). There were of course notable exceptions within these overall averages, which included BMR accounting for a higher proportions of FMR for harbor porpoise (73%), Weddell seals (86%) and Steller sea lions (73%). These results were generally due to both higher-than-average BMR and lower-than-average FMR estimates.

## Factors that Impact Non-Active Metabolic Rate

As insightful as it is to quantify differences in energy expenditure under the tightly defined conditions of BMR, it is equally important to understand how various intrinsic factors (e.g. age, sex, reproduction, molt, individual) and extrinsic factors (e.g. food availability, water temperature, season) can influence metabolism. Under these conditions, one cannot (by definition) restrict the comparisons to studies that match Kleiber's criteria, and the nomenclature becomes problematic. For this portion of the review we use the term resting metabolic rate (RMR) to designate measures of non-active (resting) energy expenditure obtained under ‘non-Kleiber conditions’.

By examining RMR outside of ‘Kleiber conditions’, we can explore the influence of intrinsic factors that can impact metabolism. As evidenced in numerous species, including northern elephant seals (Maresh *et al.,* 2014), harbor seals (*Phoca vitulina*; Rosen and Renouf, 1998), as well as the Australian fur seal (*Arctocephalus pusillus*), New Zealand fur seal (*Arctocephalus forsteri)* and Australian sea lion (*Neophoca cinerea*) (Ladds *et al.,* 2017c), we find that mass-specific RMR decreases with age as physiology matures and growth rates slow. In most pinnipeds, females have a higher mass-specific RMR than males (Boyd and Duck, 1991; Rosen and Renouf, 1995; Boyd and Croxall, 1996; Hurley and Costa, 2001; Ladds *et al.,* 2017c). Yet surprisingly, female reproduction does not appear to alter RMR, although it does alter TEE as evidenced by increased food consumption (e.g. Williams *et al.,* 2007). For example, RMR of lactating and non-lactating northern fur seals (*Callorhinus ursinus*) were similar (Costa and Gentry, 1986), as was RMR of pregnant, lactating and non-reproductive California sea lions (*Zalophus californianus*) (Williams *et al.,* 2007). Moreover, the RMR of bottlenose dolphins did not change throughout gestation (Yazdi *et al.,* 1999). In contrast, RMR of pregnant sea otters declines during gestation, and then increases significantly over the first 3 months of lactation (Thometz *et al.,* 2016).

**Figure 5:**
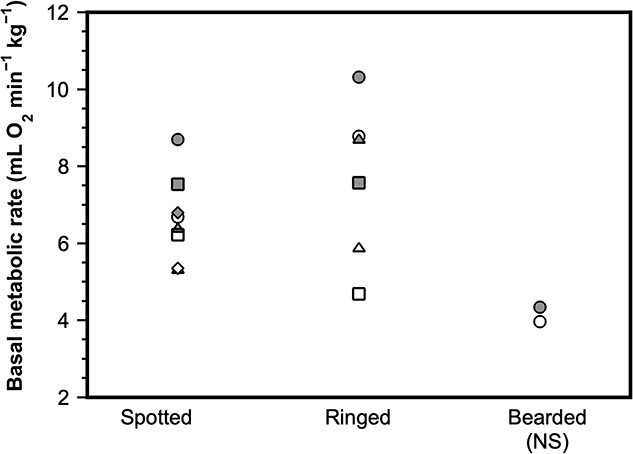
Variation in basal metabolic rate (BMR) within and across individuals for three phocid seal species. Unique symbols are used for each individual seal within each of the species, with white and black symbols denoting the molting BMR and non-molting BMR, respectively. Data is from Thometz *et al.,* 2021, who stated that intraindividual variation in BMR for all four spotted and three ringed seals was associated with the cost of the molt. Yet it is important to note that BMR variation across individuals of the same species is larger than the BMR variations observed within individuals. There was no effect of the molt on BMR of bearded seals as denoted by NS, non-significant.

The molt is another intrinsic factor that influences the basic metabolic expenditure of pinnipeds. The molt appears to increase BMR in numerous species of phocids, including the southern elephant (*Mirounga leonina*) (Boyd *et al.,* 1993), gray (*Halichoerus grypus*) (Sparling *et al.,* 2006), harbor (Paterson *et al.,* 2021), spotted (*Phoca largha*) and ringed (*Pusa hispida*) (Thometz *et al.,* 2021) seals. A similar effect is seen among otariids, including Australian sea lion, as well as the Australian, New Zealand (Ladds *et al.,* 2017b) and northern (Dalton *et al.,* 2015; McHuron *et al.,* 2019) fur seals. However, it should be noted that differences amongst the metabolism of individuals in these species can be larger than the variation in metabolism associated with the molt (Figure 5). Interestingly, the molt only increased the metabolism of non-reproductive California sea lions; it did not alter the RMR of reproductive females, which could suggest that reproductive females were constrained by a metabolic ceiling (Williams *et al.,* 2007). Also of note is that the BMR of the bearded seal (*Erignathus barbatus)*, the phocid species with the longest molt (119 days compared to approximately 30 days for many other species) does not seem to change, probably because the energetic cost of the molt is spread out over a long duration (Thometz *et al.,* 2021). In contrast, BMR decreased during the molt in harbor (Ashwell-Erickson *et al.,* 1986; Rosen and Renouf, 1998) and northern elephant (Worthy *et al.,* 1992) seals, but these animals were also fasting, which can contribute to a phenomenon called metabolic depression.

Metabolic depression is a common physiological response to either normal or unexpected periods of nutritional deprivation, typically measured as a decrease in RMR (Guppy and Withers, 1999). Its onset is thought to be associated with the shift from glycogenolysis to gluconeogenesis (phase 2 fasting; Cahill, 1978, Castellini and Rea, 1992, Cherel *et al.,* 1992). Metabolic depression has been noted in numerous phocids during natural fasting periods associated with the molt and weaning (Worthy and Lavigne, 1987; Nordøy *et al.,* 1990; Markussen, 1995), as well as during unexpected decreases in food intake (Rosen and Trites, 2002; Rosen *et al.,* 2014). A life history that includes long-term fasting conditions may translate into low FMRs (Castellini and Rea, 1992), such as observed in the intrinsically low FMR of northern elephant seals, which is 68% lower than predicted for other marine mammals (Williams *et al.,* 2020). Villegas-Amtmann *et al.* (2017) also suggested that the intrinsically low FMRs of Galapagos sea lion (*Zalophus wollebaeki*) and Galapagos fur seals (*Arctocephalus galapagoensis*) is an adaptation to living in a limited resource habitat (an extrinsic factor).

Other extrinsic factors that could impact BMR are water temperature and season. Indeed, it has been hypothesized that the main driver of the high metabolic rate of small marine mammals is elevated heat loss in water (Kanwisher and Sundnes, 1966; Yeates *et al.,* 2007), while the comparatively low expenditure of Galapagos fur seals was attributed to an adaptative response to reduce thermal stress in warm water (Costa and Trillmich, 1988). However, the apparent influence of water temperature is not consistent across species, nor can it be directly attributable to thermoregulatory costs. Three pinniped species endemic to Australian waters (Australian fur seal, New Zealand fur seal and Australian sea lion) showed disparate results in relation to natural changes in water temperature, where one species increased BMR in response to lower water temperatures, one decreased BMR, and the third had no change in BMR (Ladds *et al.,* 2017b). These disparate results may be due to confounding influences. Indeed, among pinnipeds seasonal variation in BMR in many species have been associated with the molt or changes in body mass, overall energy expenditure or food intake rather than environmental temperatures *per se*, as seen in California sea lions (Williams *et al.,* 2007), harbor seals (Rosen and Renouf, 1998), grey seals (Sparling *et al.,* 2006) and northern fur seals (Dalton *et al.,* 2015; McHuron *et al.,* 2019). Likewise, the BMR of some cetaceans, including harbor porpoise (Rojano-Doñate *et al.,* 2018) and Pacific white-sided dolphin (Rechsteiner *et al.,* 2013), appear to be unaltered by seasonal changes in water temperature.

## Factors That Impact Field Metabolic Rate

Unlike BMR, measures of FMR can encompass a wide range of behaviors and life history stages. For many marine mammals, FMR measures are made during the reproductive season, partly for scientific interest but also due to the logistical requirements for recapturing individuals. As reproduction can be an energetically expensive endeavor, care must be taken when applying these estimates to other times of the year. Even within a reproductive season for an individual species, FMR can vary tremendously depending on the target behavior. For example, FMR of breeding adult Antarctic fur seals (*Arctocephalus gazella*) is dependent on whether one is studying fasting territorial males (3.3 x Kleiber; Boyd and Duck, 1991), or lactating females during their initial postpartum fast (3.6 x Kleiber; Costa and Trillmich, 1988) or their at-sea foraging phase (6.7 x Kleiber; Costa *et al.,* 1989). For studies on lactating females, some studies report the energy expenditure over an entire at-sea on-land nursing cycle while others may only report the substantially more expensive at-sea portion (Costa *et al.,* 1991). Differences in FMR estimates for the entire nursing cycle versus only the at-sea portion are associated with the amount of time spent swimming and diving since locomotion is a major component of TEE.

Given that the cost of locomotion is a key component to consider for the TEE of animals, many researchers have investigated the cost of locomotion in marine mammals using respirometry in laboratory settings. As we mentioned previously, we included these measures as relevant alternate measures of FMR on the assumption that locomotion was a major cost of at-sea behaviour. However, the cost of swimming in some species was revealed to be much lower than FMR measured by the more traditional means of DLW. As previously noted, energy expenditure of bottlenose dolphins while swimming was 4.1 x Kleiber, while at-sea expenditure of wild dolphins measured via DLW was 7.1 x Kleiber (Figure 1). A similar difference was seen in the reported cost of swimming vs. at-sea FMR in harbor seals (2.8 vs 5.0 x Kleiber) and walrus (4.6 vs 6.0 x Kleiber), but this difference was not seen in Australian sea lion (4.8 vs 4.5 x Kleiber) (calculated from values in Appendix 2). Still, estimates of the cost of locomotion are invaluable for calculating FMR from activity budgets (e.g. Jeanniard du Dot *et al.,* 2016) and may still prove to be useful proxies if they represent the only measures of energy expenditure for many species of marine mammals.

There are also numerous studies that have measured the cost of diving in marine mammals. To illustrate the influence of submergence, California sea lions resting at the water's surface have a BMR of 2–3 x Kleiber but when they perform prolonged sedentary submergences their metabolic expenditure approached 1 x Kleiber (Hurley and Costa, 2001). Likewise, the metabolism of Weddell seals resting at the water surface were 1.6 to 1.8 x Kleiber (Castellini *et al.,* 1992; Williams *et al.,* 2004) but decreased to 1.1 x Kleiber when submerged (Williams *et al.,* 2004), which is similar to the decrease observed in Steller sea lions (Hastie *et al.,* 2007). Increased submergence time also lowers the metabolic rate of grey seals (Reed *et al.,* 1994) and three Australian otariid species (Ladds *et al.,* 2017c).

Care must be taken to not apply estimates of the cost of diving and swimming interchangeably, as the two are physiologically and energetically different. How exercise underwater impacts the metabolism of marine mammals is complicated because there is the ‘dive response’ (e.g. Scholander, 1940; Irving *et al.,* 1941; Scholander, 1963) that lowers rates of oxygen consumption (as evidenced by the studies discussed above) overlaid on top of a conflicting ‘exercise’ response that increases heart rate, which is an indicator of increased rates of oxygen consumption (Noren *et al.,* 2012). Indeed, looking at results from two odontocete species we find that oxygen consumption increased when the animal was active at depth, but oxygen consumption during submerged swimming was still lower than oxygen consumption during surface swimming (John, 2020). As the activity level (measured variously as swim speed, stroke rate, ODBA, etc.) of the diving seal or odontocete increases so does metabolic rate (Williams *et al.,* 2004; Fahlman *et al.,* 2013; Maresh *et al.,* 2014; Goundie *et al.,* 2015; Ladds *et al.,* 2017c).

This effect of activity on metabolism is the basis for using measures of body movement to predict energy expenditure. Comparisons of ODBA with energy expenditure derived from DLW techniques suggests that ODBA may be a good proxy for energy expenditure when energy costs are accounted for on a behavior-specific basis (Jeanniard du Dot *et al.,* 2016). However, ODBA can underestimate energy expenditure if changes in other physiological processes such as basal metabolism (as may occur during post-exercise recovery periods), stress, thermoregulation, specific dynamic action, reproduction or growth are not accounted for (Dalton *et al.,* 2014; Volpov *et al.,* 2016; Pagano and Williams, 2019). Meanwhile, the disparate physiological responses to submergence (decreased metabolism) and activity (increased metabolism) confounds the link between metabolic rate and any one specific at-sea behavior, such as dive depth, dive type, dive rate, % time diving, dive duration, foraging effort and trip duration. This is because at-sea behaviors vary in submergence time and exercise intensity. For example, although the FMR of California sea lions varied with dive duration and bout duration, it was primarily influenced by dive depth (McHuron *et al.,* 2018) while the FMR of other otariid species was influenced by dive depth and percent time diving (Arnould *et al.,* 1996; Costa and Gales, 2000) or time spent at sea (Villegas-Amtmann *et al.,* 2017; McHuron *et al.,* 2019). Interestingly, interspecific comparisons of energy expenditure in free-ranging otariids indicate that benthic-diving species often have higher at-sea FMRs than pelagic-foraging species, leading to the hypothesis that benthic diving is an energetically expensive strategy (Costa *et al.,* 2004).

## Summary

Estimates of energy expenditure are central to conservation efforts involving marine mammals. Metabolic rates determine both the resources required for healthy animal populations as well as the potential impacts of those animals on their ecosystems. Estimates of basal and resting metabolism typically serve as the foundation for individual-based bioenergetic models while measures of field metabolic rate are frequently used in large-scale ecosystem models to estimate total prey intake. The utility of such modelling is fundamentally tied to both the accuracy of these estimates and also their appropriate application.

This review has highlighted the need for discernment when choosing values for the metabolic rates that form the basis of bioenergetic models. First, the diverse methodologies and testing conditions used to estimate metabolism do not provide the same value. Second, marine mammals cannot be treated as one homogenous group; there are large taxa and species-specific variations in BMR, FMR and the relation between the two (BMR as a proportion of FMR). Third, there are several factors that impact the metabolic rates of marine mammals, including intrinsic (growth, size, sex and life history traits such as reproduction and the molt), extrinsic (prey availability, water temperature and season) and behavioral (swim effort, overall activity, dive depth, dive duration, bout duration, percentage of time diving, time spent at sea and dive type) factors. Moreover, the metabolic response to these different factors varies across species. Thus, one estimate of metabolic rate does not fit all, within an individual across seasons, within a species across age- and sex-class, and across taxonomic groups. Moreover, one FMR does not even fit a given species as prey distributions change seasonally and annually that require marine mammals to respond by altering at-sea behaviors. Clearly, additional research is needed to improve our understanding of what determines the metabolic rates of marine mammals. One recommendation is to expand controlled experimentation on marine mammal species housed in aquaria and research facilities. Numerous studies have demonstrated that the underlying physiology of wild and aquaria animals is fundamentally similar. However, their lifestyles and, hence, total energy expenditure, often differ. For example, estimates of FMR derived from aquaria animals likely represent minimum daily active costs for wild animals. However, it is important to note that studies with aquaria animals are still vital. Each of the factors listed as impacting metabolism can be explored in the same individuals, experiencing the same conditions and using the same methodology. We also need to continue validation work to develop alternate methods that reliably measure metabolic rate of difficult to study species. Finally, given the complexities of the interacting factors that influence metabolism, we recommend that those constructing bioenergetic models consult with a marine mammal bioenergetics specialist to ensure appropriate parametrization of the model.

## Author Contributions

SRN conceptualized the ideas and wrote the original manuscript and analyses. DASR revised the analyses and added additional ideas and concepts to the manuscript.

## Supplementary Material

Web_Material_coad077

## Data Availability

This is a review paper; data were drawn from peer-reviewed papers. The Appendixes in the supplemental data for this paper provide the data we used from the original papers. Full citation information for those papers are listed in our reference section.
