# Supplementary material for: What are the Metabolic Rates of Marine Mammals and What Factors Impact this Value: A review
Source: Conserv Physiol. 2023 Oct 2;11(1):coad077. doi: 10.1093/conphys/coad077 (PMC10545007; doi:10.1093/conphys/coad077)
Supplement: Web_Material_coad077 [file web_material_coad077.zip › Appendix 1 and 2.docx]

**Appendix 1.** Basal metabolic rate of marine mammals. Metabolism was measured via indirect calorimetry {open-flow respirometry via a dome or mask (OF) or pneumotachometer (PN) or closed circuit respirometry (CF)}. Only published data are included that included the body mass of the subjects so that metabolic rate could be standardized into kcal kg^-1^ d^-1^ and compared to multiples of Kleiber. When possible, data was reported at the level of the individual. Data can be converted to kJ kg^-1^ d^-1^ as 1 kcal is equivalent to 4.184 kJ.

| **Marine Mammal Species** | **Mass (kg)** | **BMR (kcal/kg/d)** | **BMR as Multiple of Kleiber** | **Medium**  **(air or water)** | **Method** | **Reference** |
| --- | --- | --- | --- | --- | --- | --- |
| **Odontocete: Monodontidae** |  |  |  |  |  |  |
| *Delphinapterus leucas* | 764 | 28.7 | 2.2 | water | OF | John (2020) |
| *Delphinapterus leucas* | 693 | 20.3 | 1.5 | water | OF | John (2020) |
| *Delphinapterus leucas* | 817 | 18.5 | 1.4 | water | OF | John (2020) |
| *Delphinapterus leucas* | 1341 | 13.0 | 1.1 | water | OF | Rosen and Trites (2013) |
| **Odontocete: Delphinidae** |  |  |  |  |  |  |
| *Lagenorhynchus obliquidens* | 115 | 69.3 | 3.2 | water | OF | Rechsteiner *et al.* (2013) |
| *Lagenorhynchus obliquidens* | 110 | 76.4 | 3.5 | water | OF | Rechsteiner *et al.* (2013) |
| *Orcinus orca* | 1880 | 17.2 | 1.6 | water | OF | Dunkin-McClenahan, unpubl. data^1^ |
| *Orcinus orca* | 4703 | 11.2 | 1.3 | water | CF | Kriete (1995) |
| *Orcinus orca* | 3363 | 10.9 | 1.2 | water | CF | Kriete (1995) |
| *Orcinus orca* | 1837 | 30.2 | 2.8 | water | OF | Williams *et al.* (2017) |
| *Orcinus orca* | 5318 | 8.8 | 1.1 | water | OF | Worthy *et al.* (2014) |
| *Tursiops truncatus* | 140.0 | 29.5 | 1.5 | water | OF | Feldkamp *et al.* (1987) cited in Yazdi *et al.* (1999) |
| *Tursiops truncatus* | 165.0 | 39.6 | 2.0 | water | OF | John (2020) |
| *Tursiops truncatus* | 180.0 | 36.1 | 1.9 | water | OF | John (2020) |
| *Tursiops truncatus* | 213.0 | 27.4 | 1.5 | water | OF | Karandeeva *et al.* (1973) |
| *Tursiops truncatus* | 190.7 | 30.2 | 1.6 | water | OF | van der Hoop *et al.* (2014) |
| *Tursiops truncatus* | 145.0 | 51.2 | 2.5 | water | OF | Williams *et al.* (1993) |
| *Tursiops truncatus* | 145.0 | 48.6 | 2.4 | water | OF | Williams *et al.* (1993) |
| *Tursiops truncatus* | 148.6 | 45.4 | 2.3 | water | OF | Williams *et al.* (2001) |
| *Tursiops truncatus* | 229.3 | 44.1 | 2.5 | water | OF | Williams *et al.* (2017) |
| **Odontocete: Phocenidae** |  |  |  |  |  |  |
| *Phocoena phocoena* | 33.0 | 71.5 | 2.4 | water | OF | Karandeeva *et al.* (1973) |
| **Pinniped: Otariidae** |  |  |  |  |  |  |
| *Arctocephalus forsteri* | 96.7 | 44.6 | 2.0 | water | OF | Ladds *et al.* (2017b) |
| *Arctocephalus pusillus doriferus* | 73.9 | 46.1 | 1.9 | water | OF | Ladds *et al.* (2017b) |
| *Arctocephalus pusillus doriferus* | 197.0 | 37.0 | 2.0 | water | OF | Ladds *et al.* (2017b) |
| *Arctocephaluspusillus doriferus* | 71.2 | 85.7 | 3.6 | water | OF | Ladds *et al.* (2017c) |
| *Arctocephaluspusillus doriferus* | 177.4 | 70.4 | 3.7 | water | OF | Ladds *et al.* (2017c) |
| *Arctocephaluspusillus forsteri* | 153.4 | 68.7 | 3.5 | water | OF | Ladds *et al.* (2017c) |
| *Arctocephaluspusillus forsteri* | 151.7 | 66.8 | 3.4 | water | OF | Ladds *et al.* (2017c) |
| *Eumetopias jubatus* | 215.4 | 55.6 | 3.0 | water | OF | Fahlman *et al.* (2008) |
| *Eumetopias jubatus* | 162.6 | 63.4 | 3.2 | water | OF | Fahlman *et al.* (2008) |
| *Eumetopias jubatus* | 141.8 | 68.8 | 3.4 | water | OF | Fahlman *et al.* (2008) |
| *Eumetopias jubatus* | 171.0 | 109.6 | 5.7 | water | PN | Fahlman *et al.* (2016a) |
| *Eumetopias jubatus* | 160.0 | 72.2 | 3.7 | water | PN | Fahlman *et al.* (2016a) |
| *Eumetopias jubatus* | 229.0 | 75.5 | 4.2 | water | PN | Fahlman *et al.* (2016a) |
| *Eumetopias jubatus* | 204.0 | 76.0 | 4.1 | water | PN | Fahlman *et al.* (2016a) |
| *Eumetopias jubatus* | 225.0 | 61.1 | 3.4 | water | OF | Goundie (2015) |
| *Eumetopias jubatus* | 211.0 | 54.9 | 3.0 | water | OF | Goundie (2015) |
| *Eumetopias jubatus* | 170.0 | 72.3 | 3.7 | water | OF | Goundie (2015) |
| *Eumetopias jubatus* | 152.0 | 61.1 | 3.1 | water | OF | Goundie (2015) |
| *Neophoca cinerea* | 63.2 | 51.7 | 2.1 | water | OF | Ladds *et al.* (2017b) |
| *Neophoca cinerea* | 141.4 | 41.3 | 2.0 | water | OF | Ladds *et al.* (2017b) |
| *Neophoca cinerea* | 59.0 | 88.4 | 3.5 | water | OF | Ladds *et al.* (2017c) |
| *Neophoca cinerea* | 68.0 | 78.0 | 3.2 | water | OF | Ladds *et al.* (2017c) |
| *Neophoca cinerea* | 66.9 | 87.9 | 3.6 | water | OF | Ladds *et al.* (2017c) |
| *Neophoca cinerea* | 71.6 | 68.2 | 2.8 | water | OF | Ladds *et al.* (2017c) |
| *Neophoca cinerea* | 161.4 | 68.4 | 3.5 | water | OF | Ladds *et al.* (2017c) |
| *Zalophus californianus* | 69.0 | 72.6 | 3.0 | water | OF | Hurley and Costa (2001) |
| *Zalophus californianus* | 63.0 | 69.5 | 2.8 | water | OF | Hurley and Costa (2001) |
| *Zalophus californianus* | 134.0 | 39.7 | 1.9 | water | OF | Hurley and Costa (2001) |
| *Zalophus californianus* | 121.0 | 49.6 | 2.4 | water | OF | Hurley and Costa (2001) |
| *Zalophus californianus* | 61.6 | 94.3 | 3.8 | water | OF | Liao (1990) |
| *Zalophus californianus* | 73.0 | 49.2 | 2.1 | water | OF | Liao (1990) |
| **Pinniped: Phocidae** |  |  |  |  |  |  |
| *Erignathus barbatus* | 179.0 | 19.8 | 1.0 | water | OF | Thometz *et al.* (2023) |
| *Halichoerus grypus* | 198.0 | 18.1 | 1.0 | air | OF | Boily and Lavigne (1995) |
| *Halichoerus grypus* | 189.0 | 20.3 | 1.1 | air | OF | Boily and Lavigne (1995) |
| *Halichoerus grypus* | 185.0 | 22.6 | 1.2 | air | OF | Boily and Lavigne (1995) |
| *Halichoerus grypus* | 188.7 | 20.6 | 1.1 | air | OF | Boily and Lavigne (1997) |
| *Halichoerus grypus* | 190.5 | 20.4 | 1.1 | air | OF | Boily and Lavigne (1997) |
| *Halichoerus grypus* | 202.2 | 19.2 | 1.0 | air | OF | Boily and Lavigne (1997) |
| *Halichoerus grypus* | 210.4 | 18.5 | 1.0 | air | OF | Boily and Lavigne (1997) |
| *Halichoerus grypus* | 190.0 | 28.9 | 1.5 | air | OF | Boily (1996) |
| *Halichoerus grypus* | 207.5 | 21.0 | 1.1 | air | OF | Boily (1996) |
| *Halichoerus grypus* | 160.0 | 30.0 | 1.5 | air | OF | Fedak and Anderson (1982) |
| *Halichoerus grypus* | 170.0 | 13.7 | 0.7 | water | OF | Lavigne *et al.* (1986) |
| *Halichoerus grypus* | 176.0 | 15.1 | 0.8 | water | OF | Lavigne *et al.* (1986) |
| *Halichoerus grypus* | 178.0 | 16.5 | 0.9 | water | OF | Innes (1984)^2^ |
| *Halichoerus grypus* | 172.0 | 16.2 | 0.8 | water | OF | Innes (1984) |
| *Halichoerus grypus* | 179.0 | 17.3 | 0.9 | water | OF | Innes (1984) |
| *Halichoerus grypus* | 126.0 | 46.3 | 2.2 | water | OF | Sparling *et al.* (2006) |
| *Halichoerus grypus* | 143.0 | 38.3 | 1.9 | water | OF | Sparling *et al.* (2006) |
| *Halichoerus grypus* | 148.0 | 35.0 | 1.7 | water | OF | Sparling *et al.* (2006) |
| *Halichoerus grypus* | 172.0 | 43.7 | 2.3 | water | OF | Sparling *et al.* (2006) |
| *Leptonychotes weddellii* | 355.0 | 28.5 | 1.8 | water | OF | Castellini *et al.* (1992) |
| *Leptonychotes weddellii* | 425.0 | 34.9 | 2.3 | water | OF | Kooyman *et al.* (1973) |
| *Leptonychotes weddellii* | 388.5 | 24.9 | 1.6 | water | OF | Williams *et al.* (2001) |
| *Neomonachus schauinslandi* | 198.0 | 21.8 | 1.2 | water | OF | John *et al.* (2021) |
| *Pagophilus groenlandicus* | 108.0 | 22.1 | 1.0 | water | OF | Gallivan and Ronald (1979) |
| *Pagophilus groenlandicus* | 160.0 | 16.9 | 0.9 | water | OF | Gallivan and Ronald (1979) |
| *Pagophilus groenlandicus* | 154.0 | 21.5 | 1.1 | water | OF | Gallivan and Ronald (1981) |
| *Pagophilus groenlandicus* | 95.0 | 13.2 | 0.6 | water | OF | Hedd *et al.* (1997) |
| *Pagophilus groenlandicus* | 95.0 | 18.8 | 0.8 | water | OF | Hedd *et al.* (1997) |
| *Pagophilus groenlandicus* | 105.0 | 33.9 | 1.6 | water | OF | Innes (1984)^2^ |
| *Pagophilus groenlandicus* | 105.0 | 23.4 | 1.1 | water | OF | Innes (1984) |
| *Pagophilus groenlandicus* | 118.7 | 21.3 | 1.0 | water | OF | Øritsland and Ronald (1975) |
| *Phoca largha* | 76.6 | 37.4 | 1.6 | water | OF | Thometz *et al.* (2021) |
| *Phoca largha* | 61.6 | 48.5 | 1.9 | water | OF | Thometz *et al.* (2021) |
| *Phoca largha* | 65.8 | 38.0 | 1.5 | water | OF | Thometz *et al.* (2021) |
| *Phoca vitulina* | 63.0 | 32.0 | 1.3 | water | OF | Davis *et al.* (1985) |
| *Phoca vitulina* | 116.0 | 34.3 | 1.6 | air | OF | Innes (1984)^2^ |
| *Phoca vitulina* | 98.0 | 27.4 | 1.2 | air | OF | Matsuura and Whittow (1973) |
| *Phoca vitulina* | 99.8 | 23.8 | 1.1 | water | OF | Rosen and Renouf (1998) |
| *Phoca vitulina* | 83.9 | 34.0 | 1.5 | water | OF | Rosen and Renouf (1998) |
| *Phoca vitulina* | 82.8 | 26.0 | 1.1 | water | OF | Rosen and Renouf (1998) |
| *Phoca vitulina* | 89.2 | 33.9 | 1.5 | water | OF | Rosen and Renouf (1998) |
| *Phoca vitulina* | 78.9 | 38.6 | 1.6 | water | OF | Rosen and Renouf (1998) |
| *Pusa hispida* | 59.0 | 25.8 | 1.0 | water | OF | Innes (1984)^2^ |
| *Pusa hispida* | 47.5 | 22.8 | 0.9 | water | OF | Innes (1984) |
| *Pusa hispida* | 32.0 | 22.8 | 0.8 | water | OF | Parsons (1977) ^2^ |
| *Pusa hispida* | 38.5 | 20.7 | 0.7 | water | OF | Parsons (1977) |
| *Pusa hispida* | 41.0 | 19.7 | 0.7 | water | OF | Parsons (1977) |
| *Pusa hispida* | 72.0 | 15.3 | 0.6 | water | OF | Parsons (1977) |
| *Pusa hispida* | 28.0 | 62.0 | 2.0 | water | OF | Thometz *et al.* (2021) |
| *Pusa hispida* | 28.3 | 44.3 | 1.5 | water | OF | Thometz *et al.* (2021) |
| **Pinniped: Odobenidae** |  |  |  |  |  |  |
| *Odobenus rosmarus* | 705.0 | 35.4 | 2.6 | water | OF | Borque-Espinosa *et al.* (2021) |
| *Odobenus rosmarus* | 975.0 | 39.0 | 3.1 | water | OF | Borque-Espinosa *et al.* (2021) |
| *Odobenus rosmarus* | 825.0 | 39.9 | 3.1 | water | OF | Borque-Espinosa *et al.* (2021) |
| **Mustelidae** |  |  |  |  |  |  |
| *Enhydra lutris* | 17.3 | 81.3 | 2.4 | water | OF | Costa and Kooyman (1982) |
| *Enhydra lutris* | 18.4 | 75.8 | 2.2 | water | OF | Costa and Kooyman (1984) |
| *Enhydra lutris* | 18.0 | 83.4 | 2.5 | air | CF | Morrison *et al.* (1974) |
| *Enhydra lutris* | 13.5 | 93.8 | 2.6 | water | OF | Williams (1989) |
| *Enhydra lutris* | 27.3 | 92.4 | 3.0 | water | OF | Yeates *et al.* (2007) |
| **Sirenia** |  |  |  |  |  |  |
| *Trichechus manatus* | 170.5 | 5.61 | 0.3 | water | OF | Gallivan and Best (1980) |
| *Trichechus manatus* | 82.5 | 6.59 | 0.3 | water | OF | Gallivan and Best (1980) |
| *Trichechus manatus* | 427.7 | 3.23 | 0.2 | water | OF | Irvine (1983) |
| *Trichechus manatus* | 545.0 | 9.95 | 0.7 | water | OF | John *et al.* (2021) |
| *Trichechus manatus* | 819.0 | 5.79 | 0.4 | water | OF | John *et al.* (2021) |

*1. Although measures of BMR for Orca orcinus from Dunkin-McClenahan are unpublished as a primary source, we have included it as it has been vetted by several previous researchers.*

*2. Data from theses by Innes (1984) and Parsons (1977) was subsequently republished as part of a review article in Lavigne et al. (1986).*

**Appendix 2.** Field metabolic rate (FMR) of individual marine mammals. Data were only included if it provided the body mass of the subjects so that metabolic rate could be standardized into kcal kg^-1^ d^-1^ and compared to multiples of Kleiber. Data can be converted to MJ/d/kg as 1 MJ is equivalent to 238.85 kcal. Method to measure metabolism was either doubly labeled water (DLW), measured changes in body mass (MC), open-flow respirometry (OF) or pneumotachometer (P). The types of encapsulated behaviors and life stages included FMR of lactating females for both at-sea and on-land portions of their lactation cycles (FMR-LF), the at-sea portion only (FMR-LFS), or the on-land portion only (FMR-LFL, which was usually restricted to the initial postpartum period), fasting non-lactating females (FMR-FF) breeding males (FMR-BM, which were usually fasting on-land periods), and at-sea energy expenditure of various non-breeding individuals (FMR-S). Daily energy expenditure was also measured in captive individuals (FMR-C), as was measurements of swimming costs measured either post-submerged swim (PSS) or post-submerged exercise (PSE).

|  | Mass | FMR (kcal/kg/d) | Kleiber | Method | Measure | Wild/captive | Reference |
| --- | --- | --- | --- | --- | --- | --- | --- |
| **Odontocete: Monodontidae** |  |  |  |  |  |  |  |
| *Delphinapterus leucas* | 693 | 33 | 2.4 | OF | PSS | captive | John (2020) |
| *Delphinapterus leucas* | 817 | 32 | 2.5 | OF | PSS | captive | John (2020) |
| *Delphinapterus leucas* | 764 | 55 | 4.1 | OF | PSS | captive | John (2020) |
| **Odontocete: Delphinidae** |  |  |  |  |  |  |  |
| *Tursiops truncatus* | 170 | 135 | 6.9 | DLW | FMR-S | wild | Bejarano *et al.* (2017)^2^ |
| *Tursiops truncatus* | 203 | 133 | 7.2 | DLW | FMR-S | wild | Bejarano *et al.* (2017) |
| *Tursiops truncatus* | 178.3 | 40 | 2.1 | PN | PSE | captive | Fahlman *et al.* (2016b) |
| *Tursiops truncatus* | 195.8 | 45 | 2.4 | PN | PSE | captive | Fahlman *et al.* (2016b) |
| *Tursiops truncatus* | 167.5 | 53 | 2.7 | PN | PSE | captive | Fahlman *et al.* (2016b) |
| *Tursiops truncatus* | 249.5 | 55 | 3.1 | PN | PSE | captive | Fahlman *et al.* (2016b) |
| *Tursiops truncatus* | 165 | 55 | 2.8 | OF | PSS | captive | John (2020) |
| *Tursiops truncatus* | 213.6 | 41 | 2.2 | DLW | FMR-C | captive | Rimbach *et al.* (2021) |
| *Tursiops truncatus* | 256.8 | 27 | 1.6 | DLW | FMR-C | captive | Rimbach *et al.* (2021) |
| *Tursiops truncatus* | 210 | 62 | 3.4 | DLW | FMR-C | captive | Rimbach *et al.* (2021) |
| *Tursiops truncatus* | 265.4 | 69 | 4.0 | DLW | FMR-C | captive | Rimbach *et al.* (2021) |
| *Tursiops truncatus* | 257.2 | 78 | 4.5 | DLW | FMR-C | captive | Rimbach *et al.* (2021) |
| *Tursiops truncatus* | 160.9 | 81 | 4.1 | DLW | FMR-C | captive | Rimbach *et al.* (2021) |
| *Tursiops truncatus* | 142.9 | 49 | 2.4 | DLW | FMR-C | captive | Rimbach *et al.* (2021) |
| *Tursiops truncatus* | 245.8 | 47 | 2.7 | DLW | FMR-C | captive | Rimbach *et al.* (2021) |
| *Tursiops truncatus* | 199.6 | 69 | 3.7 | DLW | FMR-C | captive | Rimbach *et al.* (2021) |
| *Tursiops truncatus* | 193 | 78 | 4.2 | DLW | FMR-C | captive | Rimbach *et al.* (2021) |
| *Tursiops truncatus* | 166-250 | 91 | 5.0 | OF | PSS | captive | van der Hoop *et al.* (2014) |
| *Tursiops truncatus* | 229 | 80 | 4.4 | OF | PSS | captive | Williams *et al.* (2017) |
| **Odontocete: Phocenidae** |  |  |  |  |  |  |  |
| *Phocoena phocoena* | 64.0 | 83 | 3.3 | DLW | FMR-C | captive | Rojano-Doñate *et al.* (2018) |
| *Phocoena phocoena* | 56.0 | 96 | 3.7 | DLW | FMR-C | captive | Rojano-Doñate *et al.* (2018) |
| *Phocoena phocoena* | 65.0 | 72 | 2.9 | DLW | FMR-C | captive | Rojano-Doñate *et al.* (2018) |
| *Phocoena phocoena* | 60.0 | 86 | 3.4 | DLW | FMR-S | wild | Rojano-Doñate *et al.* (2018)^1^ |
|  |  |  |  |  |  |  |  |
| **Pinniped: Otaridae** |  |  |  |  |  |  |  |
| *Arctocephalus forsteri* | 154 | 128 | 6.4 | OF | PSS | captive | Ladds *et al.* (2017a) |
| *Arctocephalus forsteri* | 54-55 | 240 | 9.3 | OF | PSS | captive | Ladds *et al.* (2017a) |
| *Arctocephalus forsteri* | 149-161 | 74 | 3.7 | OF | PSS | captive | Ladds *et al.* (2017a) |
| *Arctocephalus galapagoensis* | 33.2 | 51 | 1.8 | DLW | FMR – LFL | wild | Costa and Trillmich (1988) |
| *Arctocephalus galapagoensis* | 37.9 | 23 | 0.8 | DLW | FMR – LFL | wild | Costa and Trillmich (1988) |
| *Arctocephalus galapagoensis* | 41.1 | 18 | 0.7 | DLW | FMR – LFL | wild | Costa and Trillmich (1988) |
| *Arctocephalus gazella* | 36.8 | 131 | 4.6 | DLW | FMR - LFS | wild | Arnould *et al.* (1996) |
| *Arctocephalus gazella* | 188 | 63 | 3.3 | MC | FMR - BM | wild | Boyd and Duck (1991) |
| *Arctocephalus gazella* | 39.4 | 101 | 3.6 | DLW | FMR – LFL | wild | Costa and Trillmich (1988) |
| *Arctocephalus gazella* | 34.6 | 160 | 5.5 | DLW | FMR - LFS | wild | Costa *et al.* (1989) |
| *Arctocephalus gazella* | 31.0 | 141 | 4.8 | DLW | FMR - LFS | wild | Jeanniard-du-Dot *et al.* (2016) |
| *Arctocephalus pusillus* | 179-182 | 123 | 6.4 | OF | PSS | captive | Ladds *et al.* (2017a) |
| *Arctocephalus pusillus* | 69-78 | 146 | 6.1 | OF | PSS | captive | Ladds *et al.* (2017a) |
| *Arctocephalus pusillus doriferus* | 71.2 | 116 | 4.8 | OF | PSS | captive | Ladds *et al.* (2017c) |
| *Arctocephalus pusillus doriferus* | 177.4 | 106 | 5.5 | OF | PSS | captive | Ladds *et al.* (2017c) |
| *Arctocephalus pusillus forsteri* | 153.4 | 44 | 2.2 | OF | PSS | captive | Ladds *et al.* (2017c) |
| *Arctocephalus pusillus forsteri* | 151.7 | 130 | 6.5 | OF | PSS | captive | Ladds *et al.* (2017c) |
| *Arctocephalus pusillus forsteri* | 54.0 | 210 | 8.1 | OF | PSS | captive | Ladds *et al.* (2017c) |
| *Callorhinus ursinus* | 42.7 | 96 | 3.5 | DLW | FMR-LFL | wild | Costa and Gentry (1986) |
| *Callorhinus ursinus* | 43.2 | 169 | 6.2 | DLW | FMR-FF | wild | Costa and Gentry (1986) |
| *Callorhinus ursinus* | 51.1 | 170 | 6.5 | DLW | FMR - LFS | wild | Costa *et al.* (1985) |
| *Callorhinus ursinus* | 38.0 | 134 | 4.7 | DLW | FMR - LFS | wild | Jeanniard-du-Dot *et al.* (2016) |
| *Callorhinus ursinus* | 27.5 | 161 | 5.3 | DLW | FMR - LFS | wild | McHuron *et al.* (2019) |
| *Callorhinus ursinus* | 46.5 | 107 | 4.0 | DLW | FMR - LFS | wild | McHuron *et al.* (2019) |
| *Callorhinus ursinus* | 33.0 | 134 | 4.6 | DLW | FMR - LFS | wild | McHuron *et al.* (2019) |
| *Callorhinus ursinus* | 450 | 127 | 4.7 | DLW | FMR - LFS | wild | McHuron *et al.* (2019) |
| *Callorhinus ursinus* | 40.5 | 131 | 4.7 | DLW | FMR - LFS | wild | McHuron *et al.* (2019) |
| *Callorhinus ursinus* | 260 | 142 | 4.6 | DLW | FMR - LFS | wild | McHuron *et al.* (2019) |
| *Callorhinus ursinus* | 34.5 | 182 | 6.3 | DLW | FMR - LFS | wild | McHuron *et al.* (2019) |
| *Callorhinus ursinus* | 39.5 | 163 | 5.8 | DLW | FMR - LFS | wild | McHuron *et al.* (2019) |
| *Callorhinus ursinus* | 32.5 | 139 | 4.8 | DLW | FMR - LFS | wild | McHuron *et al.* (2019) |
| *Callorhinus ursinus* | 36.5 | 141 | 5.0 | DLW | FMR - LFS | wild | McHuron *et al.* (2019) |
| *Callorhinus ursinus* | 35.5 | 145 | 5.0 | DLW | FMR - LFS | wild | McHuron *et al.* (2019) |
| *Callorhinus ursinus* | 43.5 | 153 | 5.6 | DLW | FMR - LFS | wild | McHuron *et al.* (2019) |
| *Callorhinus ursinus* | 35.0 | 137 | 4.8 | DLW | FMR - LFS | wild | McHuron *et al.* (2019) |
| *Callorhinus ursinus* | 36.5 | 151 | 5.3 | DLW | FMR - LFS | wild | McHuron *et al.* (2019) |
| *Callorhinus ursinus* | 45.5 | 138 | 5.1 | DLW | FMR - LFS | wild | McHuron *et al.* (2019) |
| *Callorhinus ursinus* | 36.5 | 144 | 5.1 | DLW | FMR - LFS | wild | McHuron *et al.* (2019) |
| *Callorhinus ursinus* | 42.0 | 134 | 4.9 | DLW | FMR - LFS | wild | McHuron *et al.* (2019) |
| *Callorhinus ursinus* | 39.6 | 155 | 5.6 | DLW | FMR - LFS | wild | McHuron *et al.* (2019) |
| *Callorhinus ursinus* | 36.0 | 142 | 5.0 | DLW | FMR - LFS | wild | McHuron *et al.* (2019) |
| *Callorhinus ursinus* | 46.0 | 133 | 5.0 | DLW | FMR - LFS | wild | McHuron *et al.* (2019) |
| *Callorhinus ursinus* | 45.0 | 134 | 4.9 | DLW | FMR - LFS | wild | McHuron *et al.* (2019) |
| *Callorhinus ursinus* | 48.6 | 145 | 5.5 | DLW | FMR - LFS | wild | McHuron *et al.* (2019) |
| *Callorhinus ursinus* | 36.4 | 138 | 4.9 | DLW | FMR - LFS | wild | McHuron *et al.* (2019) |
| *Callorhinus ursinus* | 42.0 | 142 | 5.2 | DLW | FMR - LFS | wild | McHuron *et al.* (2019) |
| *Callorhinus ursinus* | 44.6 | 128 | 4.7 | DLW | FMR - LFS | wild | McHuron *et al.* (2019) |
| *Callorhinus ursinus* | 47.6 | 146 | 5.5 | DLW | FMR - LFS | wild | McHuron *et al.* (2019) |
| *Callorhinus ursinus* | 35.0 | 146 | 5.1 | DLW | FMR - LFS | wild | McHuron *et al.* (2019) |
| *Callorhinus ursinus* | 36.0 | 140 | 4.9 | DLW | FMR - LFS | wild | McHuron *et al.* (2019) |
| *Callorhinus ursinus* | 34.0 | 136 | 4.7 | DLW | FMR - LFS | wild | McHuron *et al.* (2019) |
| *Callorhinus ursinus* | 37.2 | 140 | 5.0 | DLW | FMR - LFS | wild | McHuron *et al.* (2019) |
| *Callorhinus ursinus* | 35.6 | 154 | 5.4 | DLW | FMR - LFS | wild | McHuron *et al.* (2019) |
| *Callorhinus ursinus* | 35.0 | 154 | 5.3 | DLW | FMR - LFS | wild | McHuron *et al.* (2019) |
| *Callorhinus ursinus* | 44.6 | 114 | 4.2 | DLW | FMR - LFS | wild | McHuron *et al.* (2019) |
| *Callorhinus ursinus* | 42.4 | 157 | 5.7 | DLW | FMR - LFS | wild | McHuron *et al.* (2019) |
| *Callorhinus ursinus* | 34.0 | 114 | 3.9 | DLW | FMR - LFS | wild | McHuron *et al.* (2019) |
| *Callorhinus ursinus* | 34.2 | 165 | 5.7 | DLW | FMR - LFS | wild | McHuron *et al.* (2019) |
| *Callorhinus ursinus* | 34.0 | 114 | 3.9 | DLW | FMR - LFS | wild | McHuron *et al.* (2019) |
| *Callorhinus ursinus* | 34.4 | 146 | 5.0 | DLW | FMR - LFS | wild | McHuron *et al.* (2019) |
| *Callorhinus ursinus* | 34.2 | 122 | 4.2 | DLW | FMR - LFS | wild | McHuron *et al.* (2019) |
| *Callorhinus ursinus* | 33.4 | 134 | 4.6 | DLW | FMR - LFS | wild | McHuron *et al.* (2019) |
| *Callorhinus ursinus* | 45.8 | 151 | 5.6 | DLW | FMR - LFS | wild | McHuron *et al.* (2019) |
| *Callorhinus ursinus* | 39.2 | 140 | 5.0 | DLW | FMR - LFS | wild | McHuron *et al.* (2019) |
| *Callorhinus ursinus* | 37.4 | 142 | 5.0 | DLW | FMR - LFS | wild | McHuron *et al.* (2019) |
| *Callorhinus ursinus* | 40.6 | 143 | 5.2 | DLW | FMR - LFS | wild | McHuron *et al.* (2019) |
| *Callorhinus ursinus* | 42.0 | 200 | 7.3 | DLW | FMR - LFS | wild | McHuron *et al.* (2019) |
| *Callorhinus ursinus* | 33.0 | 156 | 5.3 | DLW | FMR - LFS | wild | McHuron *et al.* (2019) |
| *Callorhinus ursinus* | 34.6 | 156 | 5.4 | DLW | FMR - LFS | wild | McHuron *et al.* (2019) |
| *Eumetopias jubatus* | 150-229 | 125 | 6.6 | OF | PSS | captive | Goundie *et al.* (2015) |
| *Eumetopias jubatus* | 150-229 | 103 | 5.5 | OF | PSS | captive | Goundie *et al.* (2015) |
| *Eumetopias jubatus* | 155-160 | 73 | 3.7 | OF | PSS | captive | Ladds *et al.* (2017a) |
| *Eumetopias jubatus* | 172-175 | 83 | 4.3 | OF | PSS | captive | Ladds *et al.* (2017a) |
| *Eumetopias jubatus* | 230-233 | 66 | 3.7 | OF | PSS | captive | Ladds *et al.* (2017a) |
| *Eumetopias jubatus* | 214-218 | 72 | 4.0 | OF | PSS | captive | Ladds *et al.* (2017a) |
| *Neophoca cinerea* | 76.5 | 146 | 6.1 | DLW | FMR - LFS | wild | Costa and Gales (2003) |
| *Neophoca cinerea* | 76.5 | 55 | 2.3 | DLW | FMR - LFS | wild | Costa and Gales (2003) |
| *Neophoca cinerea* | 83.5 | 114 | 4.9 | DLW | FMR - LFL | wild | Costa *et al.* (1991) |
| *Neophoca cinerea* | 66.0 | 155 | 6.3 | OF | PSS | captive | Ladds *et al.* (2017a) |
| *Neophoca cinerea* | 47.0 | 204 | 7.6 | OF | PSS | captive | Ladds *et al.* (2017a) |
| *Neophoca cinerea* | 153-160 | 107 | 5.4 | OF | PSS | captive | Ladds *et al.* (2017a) |
| *Neophoca cinerea* | 110-125 | 117 | 5.5 | OF | PSS | captive | Ladds *et al.* (2017a) |
| *Neophoca cinerea* | 71.6 | 55 | 2.3 | OF | PSS | captive | Ladds *et al.* (2017c) |
| *Neophoca cinerea* | 68.0 | 67 | 2.7 | OF | PSS | captive | Ladds *et al.* (2017c) |
| *Neophoca cinerea* | 59.0 | 78 | 3.1 | OF | PSS | captive | Ladds *et al.* (2017c) |
| *Neophoca cinerea* | 110 | 77 | 3.6 | OF | PSS | captive | Ladds *et al.* (2017c) |
| *Neophoca cinerea* | 66.9 | 110 | 4.5 | OF | PSS | captive | Ladds *et al.* (2017c) |
| *Neophoca cinerea* | 161.4 | 105 | 5.4 | OF | PSS | captive | Ladds *et al.* (2017c) |
| *Neophoca cinerea* | 45.0 | 185 | 6.9 | OF | PSS | captive | Ladds *et al.* (2017c) |
| *Zalophus californianus* | 84.4 | 142 | 6.1 | DLW | FMR - LFS | wild | Costa *et al.* (1991) |
| *Zalophus californianus* | 72.5 | 161 | 6.7 | DLW | FMR - LFS | wild | Costa *et al.* (1991) |
| *Zalophus californianus* | 76.8 | 163 | 6.9 | DLW | FMR - LFS | wild | Costa *et al.* (1991) |
| *Zalophus californianus* | 84.1 | 108 | 4.7 | DLW | FMR - LFS | wild | Costa *et al.* (1991) |
| *Zalophus californianus* | 76.8 | 145 | 6.1 | DLW | FMR - LFS | wild | Costa *et al.* (1991) |
| *Zalophus californianus* | 84.1 | 101 | 4.4 | DLW | FMR - LFS | wild | Costa *et al.* (1991) |
| *Zalophus californianus* | 83.3 | 68 | 2.9 | DLW | FMR - LFS | wild | McHuron *et al.* (2018) |
| *Zalophus californianus* | 61.6 | 123 | 4.9 | DLW | FMR - LFS | wild | McHuron *et al.* (2018) |
| *Zalophus californianus* | 78.9 | 144 | 6.1 | DLW | FMR - LFS | wild | McHuron *et al.* (2018) |
| *Zalophus californianus* | 81.1 | 110 | 4.7 | DLW | FMR - LFS | wild | McHuron *et al.* (2018) |
| *Zalophus californianus* | 90.6 | 124 | 5.5 | DLW | FMR - LFS | wild | McHuron *et al.* (2018) |
| *Zalophus californianus* | 79.5 | 102 | 4.4 | DLW | FMR - LFS | wild | McHuron *et al.* (2018) |
| *Zalophus californianus* | 89.3 | 133 | 5.8 | DLW | FMR - LFS | wild | McHuron *et al.* (2018) |
| *Zalophus californianus* | 78.3 | 108 | 4.6 | DLW | FMR - LFS | wild | McHuron *et al.* (2018) |
| *Zalophus californianus* | 82.5 | 81 | 3.5 | DLW | FMR - LFS | wild | McHuron *et al.* (2018) |
| *Zalophus californianus* | 74.8 | 113 | 4.7 | DLW | FMR - LFS | wild | McHuron *et al.* (2018) |
| *Zalophus californianus* | 78.4 | 107 | 4.6 | DLW | FMR - LFS | wild | McHuron *et al.* (2018) |
| *Zalophus californianus* | 76.7 | 109 | 4.6 | DLW | FMR - LFS | wild | McHuron *et al.* (2018) |
| *Zalophus californianus* | 85.4 | 98 | 4.3 | DLW | FMR - LFS | wild | McHuron *et al.* (2018) |
| *Zalophus californianus* | 81.0 | 93 | 4.0 | DLW | FMR - LFS | wild | McHuron *et al.* (2018) |
| *Zalophus californianus* | 83.3 | 108 | 4.7 | DLW | FMR - LFS | wild | McHuron *et al.* (2018) |
| *Zalophus wollebaeki* | 75.6 | 141 | 5.9 | DLW | FMR - LFS | wild | Villegas-Amtmann *et al.* (2017) |
| *Zalophus wollebaeki* | 94.8 | 107 | 4.8 | DLW | FMR - LFS | wild | Villegas-Amtmann *et al.* (2017) |
| *Zalophus wollebaeki* | 70.1 | 114 | 4.7 | DLW | FMR - LFS | wild | Villegas-Amtmann *et al.* (2017) |
| *Zalophus wollebaeki* | 73.2 | 125 | 5.2 | DLW | FMR - LFS | wild | Villegas-Amtmann *et al.* (2017) |
| *Zalophus wollebaeki* | 82.2 | 119 | 5.1 | DLW | FMR - LFS | wild | Villegas-Amtmann *et al.* (2017) |
| *Zalophus wollebaeki* | 75.1 | 114 | 4.8 | DLW | FMR - LFS | wild | Villegas-Amtmann *et al.* (2017) |
| *Zalophus wollebaeki* | 75.7 | 128 | 5.4 | DLW | FMR - LFS | wild | Villegas-Amtmann *et al.* (2017) |
| *Zalophus wollebaeki* | 65.0 | 111 | 4.5 | DLW | FMR - LFS | wild | Villegas-Amtmann *et al.* (2017) |
| *Zalophus wollebaeki* | 63.2 | 126 | 5.1 | DLW | FMR - LFS | wild | Villegas-Amtmann *et al.* (2017) |
| *Zalophus wollebaeki* | 74.8 | 121 | 5.1 | DLW | FMR - LFS | wild | Villegas-Amtmann *et al.* (2017) |
|  |  |  |  |  |  |  |  |
| **Pinniped: Phocidae** |  |  |  |  |  |  |  |
| *Cystophora cristata* | 312 | 70 | 4.2 | MC | FMR – BM | wild | Kovacs *et al.* (1996) |
| *Halichoerus grypus* | 240 | 83 | 4.6 | MC | FMR – BM | wild | Anderson and Fedak (1985) |
| *Halichoerus grypus* | 131.4 | 29 | 1.4 | DLW | FMR-C | captive | Sparling *et al.* (2008) |
| *Halichoerus grypus* | 134.6 | 34 | 1.7 | DLW | FMR-C | captive | Sparling *et al.* (2008) |
| *Halichoerus grypus* | 131.8 | 40 | 1.9 | DLW | FMR-C | captive | Sparling *et al.* (2008) |
| *Halichoerus grypus* | 128.4 | 27 | 1.3 | DLW | FMR-C | captive | Sparling *et al.* (2008) |
| *Halichoerus grypus* | 98.6 | 40 | 1.8 | DLW | FMR-C | captive | Sparling *et al.* (2008) |
| *Halichoerus grypus* | 98 | 32 | 1.5 | DLW | FMR-C | captive | Sparling *et al.* (2008) |
| *Halichoerus grypus* | 111.6 | 56 | 2.6 | DLW | FMR-C | captive | Sparling *et al.* (2008) |
| *Halichoerus grypus* | 118 | 53 | 2.5 | DLW | FMR-C | captive | Sparling *et al.* (2008) |
| *Halichoerus grypus* | 124 | 40 | 1.9 | DLW | FMR-C | captive | Sparling *et al.* (2008) |
| *Leptonychotes weddellii* | 372 | 72 | 4.5 | MC | FMR – BM | wild | Bartsh *et al.* (1992) |
| *Leptonychotes weddellii* | 355 | 24 | 1.5 | OF | FMR-S | wild | Castellini *et al.* (1992) |
| *Leptonychotes weddellii* | 355 | 34.74 | 2.2 | OF | FMR-S | wild | Castellini *et al.* (1992) |
| *Leptonychotes weddellii* | 425 | 29 | 1.9 | OF | FMR-S | wild | Kooyman *et al.* (1973) |
| *Leptonychotes weddellii* | 355 | 25 | 1.5 | OF | FMR-S | wild | Kooyman *et al.* (1980) |
| *Leptonychotes weddellii* | 263 | 18 | 1.0 | OF | FMR-S | wild | Kooyman *et al.* (1983) |
| *Leptonychotes weddellii* | 263 | 6948 | 1.6 | OF | FMR-S | wild | Kooyman *et al.* (1983) |
| *Leptonychotes weddellii* | 350 | 31 | 1.9 | OF | FMR-S | wild | Ponganis *et al.* (1993) |
| *Mirounga angustirostris* | 376 | 18 | 1.1 | DLW | FMR-S | wild | Maresh (2014) |
| *Mirounga angustirostris* | 212 | 19 | 1.0 | DLW | FMR-S | wild | Maresh *et al.* (2014) |
| *Mirounga angustirostris* | 158.2 | 29 | 1.5 | DLW | FMR-S | wild | Maresh *et al.* (2014) |
| *Neomonachus schauinslandi* | 198 | 52 | 2.8 | OF | PSS | captive | John (2020) |
| *Neomonachus schauinslandi* | 97.0 | 79 | 3.5 | OF | PSS | captive | John (2020) |
| *Phoca vitulina* | 87.5 | 53 | 2.3 | MC | FMR - LF | wild | Bowen *et al.* (1992) |
| *Phoca vitulina* | 107.5 | 87 | 4.0 | DLW | FMR - BM | wild | Coltman *et al.* (1998) |
| *Phoca vitulina* | 63.0 | 68 | 2.8 | OF | PSS | captive | Davis *et al.* (1985) |
| *Phoca vitulina* | 93.0 | 135 | 6.0 | DLW | FMR - BM | wild | Reilly and Fedak (1991) |
|  |  |  |  |  |  |  |  |
| **Piniped: Odobenidae** |  |  |  |  |  |  |  |
| *Odobenus rosmarus* | 1370 | 60 | 5.2 | DLW | FMR - BM | wild | Acquarone *et al.* (2006) |
| *Odobenus rosmarus* | 1250 | 80 | 6.8 | DLW | FMR - BM | wild | Acquarone *et al.* (2006) |
| *Odobenus rosmarus* | 705 | 39 | 2.8 | OF | PSS | captive | Borque-Espinosa *et al.* (2021) |
| *Odobenus rosmarus* | 975 | 40 | 3.2 | OF | PSS | captive | Borque-Espinosa *et al.* (2021) |
| *Odobenus rosmarus* | 825 | 44 | 3.3 | OF | PSS | captive | Borque-Espinosa *et al.* (2021) |
| *Odobenus rosmarus* | 456 | 94 | 6.2 | OF | PSS | captive | Rosen (2020) |
| *Odobenus rosmarus* | 363 | 116 | 7.2 | OF | PSS | captive | Rosen (2020) |
|  |  |  |  |  |  |  |  |
| **Mustelidae** |  |  |  |  |  |  |  |
| *Enhydra lutris* | 27.3 | 137 | 4.5 | OF | FMR - BM | wild | Yeates *et al.* (2007)^3^ |

*1. FMR of wild individual Phocoena phocoena by* Rojano-Doñate *et al.* (2018) *extrapolated from the relationship between respiration rates and energy expenditure of captive animals as determined via DLW.*

*2. Measures of FMR via doubly labelled water for Tursiops truncatus published in Bejarano et al. (2017) were originally presented by several co-authors as Costa, D.P., Worthy, G.A.J., Wells, R.S., Read, A.J., Waples, D., Scott, M.D., 2013. Patterns of seasonal metabolic rate variation for bottlenose dolphins in Sarasota Bay, Florida. In: 20th Biennial Conference on the Biology of Marine Mammals, Dunedin, New Zealand, 9–13 December 2012, p.49.*

*3. FMR of wild individual sea otters extrapolated by Yeates et al. (2007) from measured (via OF) behavior-specific metabolism of captive animals and behavior budgets of wild animals.*
